# The Rattlesnake W Chromosome: A GC-Rich Retroelement Refugium with Retained Gene Function Across Ancient Evolutionary Strata

**DOI:** 10.1093/gbe/evac116

**Published:** 2022-07-22

**Authors:** Drew R Schield, Blair W Perry, Daren C Card, Giulia I M Pasquesi, Aundrea K Westfall, Stephen P Mackessy, Todd A Castoe

**Affiliations:** Department of Ecology and Evolutionary Biology, University of Colorado, Boulder, Colorado, USA; Department of Biology, University of Texas at Arlington, Arlington, Texas, USA; School of Biological Sciences, Washington State University, Pullman, Washington, USA; Department of Organismic and Evolutionary Biology, Harvard University, Cambridge, Massachusetts, USA; Museum of Comparative Zoology, Harvard University, Cambridge, Massachusetts, USA; Department of Molecular, Cellular, and Developmental Biology, University of Colorado, Boulder, Colorado, USA; Department of Biology, University of Texas at Arlington, Arlington, Texas, USA; School of Biological Sciences, University of Northern Colorado, Greeley, Colorado, USA; Department of Biology, University of Texas at Arlington, Arlington, Texas, USA

**Keywords:** *Crotalus*, recombination suppression, snakes, transposable elements, sex chromosomes, ZW sex determination

## Abstract

Sex chromosomes diverge after the establishment of recombination suppression, resulting in differential sex-linkage of genes involved in genetic sex determination and dimorphic traits. This process produces systems of male or female heterogamety wherein the Y and W chromosomes are only present in one sex and are often highly degenerated. Sex-limited Y and W chromosomes contain valuable information about the evolutionary transition from autosomes to sex chromosomes, yet detailed characterizations of the structure, composition, and gene content of sex-limited chromosomes are lacking for many species. In this study, we characterize the female-specific W chromosome of the prairie rattlesnake (*Crotalus viridis*) and evaluate how recombination suppression and other processes have shaped sex chromosome evolution in ZW snakes. Our analyses indicate that the rattlesnake W chromosome is over 80% repetitive and that an abundance of GC-rich mdg4 elements has driven an overall high degree of GC-richness despite a lack of recombination. The W chromosome is also highly enriched for repeat sequences derived from endogenous retroviruses and likely acts as a “refugium” for these and other retroelements. We annotated 219 putatively functional W-linked genes across at least two evolutionary strata identified based on estimates of sequence divergence between Z and W gametologs. The youngest of these strata is relatively gene-rich, however gene expression across strata suggests retained gene function amidst a greater degree of degeneration following ancient recombination suppression. Functional annotation of W-linked genes indicates a specialization of the W chromosome for reproductive and developmental function since recombination suppression from the Z chromosome.

**Significance**

We report the first detailed analysis of the female-specific W chromosome in a snake. Our findings highlight distinctive features of W chromosome structure and function that provide expanded perspectives on transposable element evolution and the survival of genes related to female fitness.

## Introduction

Sex chromosomes evolve from ancestral autosomes following the establishment of sex-determining genes and the onset of recombination suppression between the newly sex-linked genomic regions (Ohno 1967; Charlesworth 1996; Bergero and Charlesworth 2009). Over time, the absence of recombination between the sex chromosomes, that is Z and W or X and Y chromosomes in female- and male-heterogametic species, respectively, often leads to an accumulation of deleterious mutations and degeneration of the sex-limited chromosomes (Charlesworth and Charlesworth 2000; Charlesworth et al. 2005; Wright et al. 2016; Abbott et al. 2017). Recombination suppression does not necessarily occur simultaneously across the entire length of the newly formed sex chromosomes (Lahn and Page 1999; Handley et al. 2004; Charlesworth et al. 2005; Matsubara et al. 2006; Nam and Ellegren 2008; Vicoso et al. 2013). Instead, chromosomal regions can experience recombination suppression at various stages during the evolutionary transition from autosomes to sex chromosomes, leading to the establishment of “evolutionary strata” that bear the signatures of this step-wise transition.

Low effective population size relative to autosomes and the lack of recombination renders selection against deleterious mutations inefficient on sex-limited chromosomes (e.g., Y or W), often resulting in an accumulation of repetitive content (Graves 2006; Bachtrog 2013). The accumulation of repeats, associated decay and loss of coding genes, and the high degree of heterochromatism on sex-limited chromosomes has promoted the view that they are genetic “wastelands” that lack major functional roles outside of sex-determination (Bachtrog 2020). However, accumulating evidence from model systems indicates that sex-limited chromosomes may play prominent additional roles in shaping sex-specific traits (Piergentili 2010; Larson et al. 2018) and have broad effects on heterochromatin state and gene regulation across the genome (Jiang et al. 2010; Brown et al. 2020a; Nguyen and Bachtrog 2021). Potential deleterious regulatory effects of sex-limited chromosome degeneration led to the “toxic-Y” hypothesis in *Drosophila* (Marais et al. 2018; Brown et al. 2020b; Wei et al. 2020), whereby the high Y-linked mutational load may correlate with the decay of physiological function and shorter lifespans in males (Sultanova et al. 2020). As a major component of this mutational load, studies have observed a disproportionate abundance of full-length and potentially transpositionally-active transposable elements (TEs) present on the sex-limited chromosomes of both XY and ZW systems (Steinemann and Steinemann 1992; Bachtrog 2003; Peona et al. 2021). Indeed, the presence of potentially active retroviral-like elements (e.g., endogenous retroviruses [ERVs]) in bird species with different degrees of W chromosome degeneration spurred the development of the “refugium hypothesis” (Peona et al. 2021), which predicts that the female-specific W chromosome, and male-specific Y chromosome in XY species, represent refugia for retroelements that may have disruptive impacts on heterochromatin formation and gene regulation.

Even in the midst of substantial genetic decay, sex-limited chromosomes may sometimes retain or acquire protein-coding genes, and in some cases may experience an amplification of specific genes through duplication events (Mank et al. 2014). These genes may be dosage-sensitive, underlie sex-specific functions, and/or be involved in meiotic drives and other forms of sexual conflict (Meiklejohn and Tao 2010; Parsch and Ellegren 2013). An associated prediction is that duplications evolve to compensate for lower overall transcription across heterochromatic sex-limited regions or because they confer sex-specific fitness advantages (Bachtrog 2020). For example, the neo-Y chromosome of *Drosophila miranda* has experienced a massive accumulation of genes that are dosage-sensitive or involved in testis-specific functions via duplication events (Bachtrog et al. 2019; Ellison and Bachtrog 2019). In mice, the Y chromosome is gene-rich due in large part to a parallel accumulation of genes that distort X chromosome transmission and restore equal population sex ratios when amplified on the Y chromosome, and which are necessary for male fertility (Soh et al. 2014; Kruger et al. 2019). Similarly, the avian W chromosome has experienced an amplification of the *HINTW* gene hypothesized to play roles in oogenesis and female fecundity (Ceplitis and Ellegren 2004). In addition to the amplification of gene products, gene duplication on sex-limited chromosomes can facilitate gene conversion events (Backström et al. 2005), presenting an evolutionary solution to guard against deleterious mutation accumulation impacting critical genes.

**Fig. 1.**
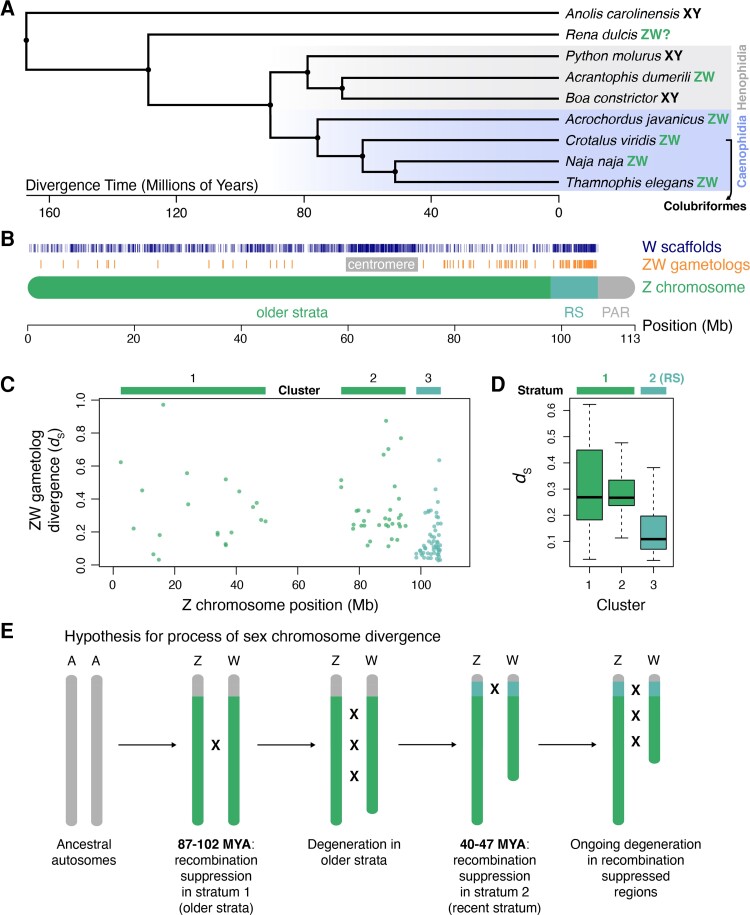
Genetic sex determination in squamates, homology between prairie rattlesnake Z and W chromosomes and the presence of evolutionary strata. (*A*) Phylogeny of representative snake species and *Anolis* outgroup with known (or presumed) genetic sex determination systems. Henophidian and caenophidian clades are highlighted. The bracketed arrow indicates species in the Colubriformes subclade of Caenophidia. Branches are scaled by divergence times in millions of years. (*B*) Locations of homologous W chromosome scaffolds and annotated coding genes (i.e., ZW gametologs) on the Z chromosome. The differently shaded sections of the Z chromosome ideogram represent distinct regions identified in Schield et al. (2019); older strata, RS = recent stratum, PAR = pseudoautosomal region. The approximate location of the centromere is labeled. (*C*) Synonymous divergence (*d*_S_) between Z and W gametologs anchored to Z chromosome positions. The three shaded regions correspond to spatial clusters of gametologs with similar levels of divergence. (*D*) Distributions of *d*_S_ by cluster with assignment to hypothesized evolutionary strata. (*E*) Schematic of the hypothesized order of recombination suppression events among evolutionary strata between the Z and W chromosomes. The approximate dates for each event are based on mean divergence estimates per stratum.

Among amniotes, snakes are a valuable model system for studying sex chromosome evolution, as male and female heterogamety has evolved independently in different lineages (fig. 1*A*; Matsubara et al. 2006, 2019; Gamble et al. 2017; Augstenová et al. 2018), providing useful comparisons to established XY and ZW model systems. Caenophidian snakes *sensu*Zaher et al. (2019), including the Acrochordoidea and Colubriformes (colubrids, elapids, viperids, and relatives), possess homologous ZW chromosomes (Rovatsos et al. 2018; fig. 1*A*). Analyses of this group are therefore of particular interest because they can provide insight into the evolutionary consequences of female heterogamety in a sex chromosome system that evolved independently of ZW chromosomes in birds (i.e., avian and caenophidian ZW chromosomes were derived from different ancestral autosomes; Matsubara et al. 2006; O’Meally et al. 2012). Despite cytogenetic studies on snake sex chromosomes beginning decades ago (e.g., Becak et al. 1964; Ohno 1967), far less is known about snake sex chromosome structure, function, and evolution compared with mammal and bird systems. Previous studies have demonstrated variation in the degree of divergence between Z and W chromosomes among caenophidian snake lineages (Matsubara et al. 2006), the presence of multiple evolutionary strata on the Z chromosome, and a lack of global dosage compensation in females (Vicoso et al. 2013; Yin et al. 2016; Schield et al. 2019). One of the hypothesized evolutionary strata on the Z chromosome exhibits multiple signatures of relatively recent recombination suppression (i.e., the so-called “recent stratum” described in Schield et al. 2019), including a lesser degree of degeneration than older strata and high levels of female-specific heterozygosity. The recent stratum may be unique to pitvipers (it was detected in *Deinagkistrodon*, *Sistrurus*, and *Crotalus* species), although additional comparative analyses and divergence time estimates to confirm the absence of recombination suppression in this region in other lineages have not been conducted. Additionally, while a previous study (Yin et al. 2016) estimated the oldest stratum to be at least 66.9 million years old, this estimate was based on a limited sampling of ZW gametologs, and the ages of more recent recombination suppression events remain unknown.

Despite progress, our understanding of the structure and composition of the snake W chromosome remains far from complete, and represents a major gap in our understanding of sex chromosome evolution across ZW systems in vertebrates. Previous investigations have been limited due primarily to the difficulty in reconstructing the W chromosome, which is the result of the heterochromatic and repetitive complexity, along with hemizygosity of the chromosome in females leading to lower genome sequencing coverage. This has in turn limited inferences regarding the number and age of evolutionary strata, repeat and gene content, and whether phenomena observed in other systems (e.g., the toxic-Y and refugium hypotheses) are also relevant in snake sex chromosome evolution. However, developments in sequencing technologies, such as linked-read sequencing (Weisenfeld et al. 2017), have improved the ability to assemble scaffolds spanning repeat-rich genomic regions. In this study, we use this technology to reconstruct the prairie rattlesnake (*Crotalus viridis*) W chromosome, and use these data to complement previously assembled genome data for autosomes and the Z chromosome of this species (Schield et al. 2019). We use these assemblies to characterize gene and repeat content on sex chromosomes to address several outstanding or otherwise poorly-resolved questions in snake sex chromosome evolution: 1) how many evolutionary strata are present between Z and W chromosomes and how long ago did recombination suppression occur, 2) what is the repeat and nucleotide composition of the W chromosome, 3) does the W chromosome represent a refugium for retroviral-like elements, as observed in other taxa, 4) do W-linked genes show evidence of retained function across evolutionary strata, and 5) is there evidence of W-specific gene duplications that may be relevant to female-specific traits or fitness?

## Results

### Evolutionary Strata on the Rattlesnake Sex Chromosomes

We identified a total of 23.7 Mb of W chromosome sequence from an assembly of a female prairie rattlesnake, represented by 2,027 scaffolds (table 1; supplementary tables S1 and S2, Supplementary Material online) assembled using high-quality 10× Genomics linked-reads (supplementary fig. S1, Supplementary Material online). Of these, 1,113 scaffolds (13.9 Mb) could be confidently anchored to the Z chromosome based on sequence homology (fig. 1*B*). The density of homologous regions is highest in the previously identified recent stratum and the centromere, matching sequence-based evidence of ZW homology in the recent stratum (Schield et al. 2019) and cytological evidence for the relative position of the viperid snake W centromere (Baker et al. 1972; Matsubara et al. 2006). There is also evidence of ZW homology across the length of the Z chromosome, albeit at lower levels, indicating more substantial W chromosome degeneration in these regions. We annotated 219 genes across these W chromosome scaffolds.

**Table 1:** W Chromosome Assembly and Annotation Statistics

Assembly	10× Supernova	10× Supernova + Agouti
**Number of scaffolds**	2,139	2,027
**Scaffold N50 (bp)**	12,080	13,252
**Longest scaffold (bp)**	62,716	153,158
**Annotated genes**	213	219

We calculated divergence between Z and W gametologs to investigate the presence of evolutionary strata and to date the onset of recombination suppression. Synonymous divergence, *d*_S_, varies considerably (mean *d*_S_ = 0.24 ± 0.19), with gametologs falling into three spatial clusters along the Z chromosome (fig. 1*C*). While there is considerable variation in *d*_S_ estimates for gametologs within clusters, broad differences in *d*_S_ among clusters suggest differential timing of recombination suppression between Z and W chromosomes across these regions (fig. 1*D*; supplementary table S3, Supplementary Material online). We used these differences to infer the approximate locations of at least two evolutionary strata between the Z and W chromosomes, supporting previous hypotheses based on studies in other viperid species (Vicoso et al. 2013; Yin et al. 2016; Schield et al. 2019). Genes in clusters 1 and 2 correspond to older strata across the majority of the Z chromosome (and there may be multiple undetectable strata across this broad region), with the highest *d*_S_ values on average (mean *d*_S_ = 0.33 ± 0.2). Cluster 3 corresponds to the recent stratum and contains genes with significantly lower *d*_S_ than the older strata (mean *d*_S_ = 0.16 ± 0.12; Mann–Whitney *U* test; *P*-value <0.0001), although we note that *d*_S_ distributions between strata do not have equal variance (Levene’s test, *P* < 2.2×10^−16^). Using lineage-specific mutation rate estimates derived from synonymous and 4-fold degenerate sites (see Methods), we transformed mean *d*_S_ values to divergence times to determine an approximate date range of recombination suppression in the older and recent strata. Based on this, we estimate that recombination suppression occurred approximately 87–102 Ma in the older strata and 40–47 Ma in the recent stratum (Fig. 1*E*).

**Fig. 2.**
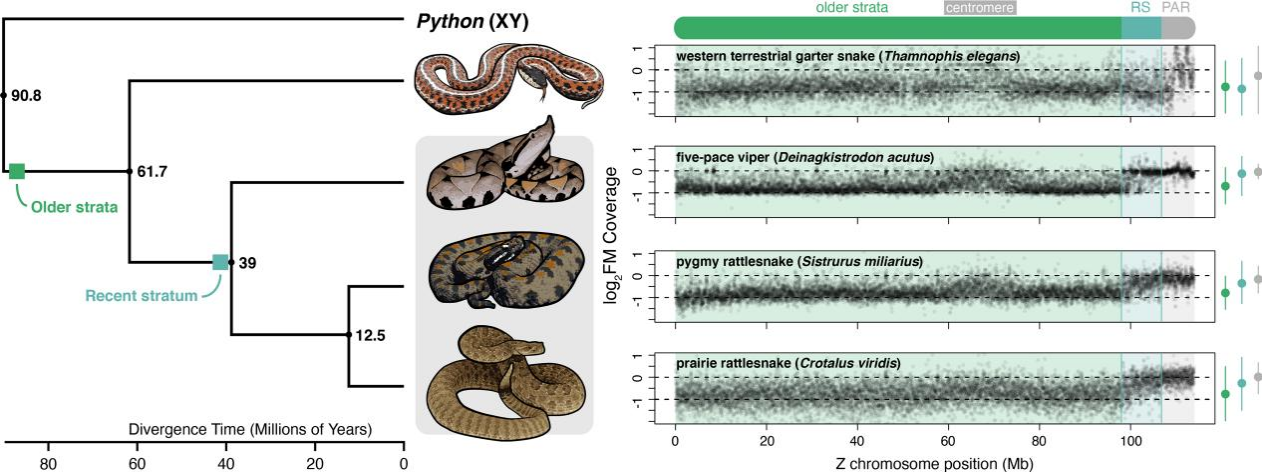
Relative female coverage across the Z chromosome in four colubroid snake species, including a representative colubrid (western terrestrial garter snake) and three pitvipers (five-pace viper, pygmy rattlesnake, and prairie rattlesnake). The phylogeny and divergence times for the four species and *Python* are shown to the left, with the grey box denoting pitviper species. Shaded boxes depict approximate dates for the formation of the older and recent strata. Panels to the right for each species show variation in the normalized ratio of female:male sequencing read coverage (log_2_FM) in 10 kb sliding windows. Horizontal dashed lines at 0 and –1 correspond to expectations for autosomal and Z-linked regions in females, respectively, and the pseudoautosomal region (PAR) and evolutionary strata (RS = recent stratum) are shaded according to the above Z chromosome ideogram. Distributions of log_2_FM in each region are summarized at the far right; points represent median log_2_FM and whiskers represent Q1–1.5×IQR and Q3 + 1.5×IQR. Images were modified from photographs from: Steve Jurvetson (garter snake), Alex White (five-pace viper), Peter Paplanus (pygmy rattlesnake), and Blair Perry (prairie rattlesnake) under Creative Commons license CC BY-NC-SA 2.0.

Comparative analyses using mapped reads across the Z chromosome from female and male garter snakes (a distantly related species with ZW sex chromosomes) indicate relative female coverage (log_2_FM) values consistently near –1 (log_2_FM = –0.81 ± 0.54) across the recent stratum (fig. 2). In contrast, each of the pitviper species analyzed has log_2_FM values in the recent stratum that are intermediate between older strata and the PAR (fig. 2). These results suggest that recombination suppression in this region occurred independently in the garter snake and pitviper lineages since their divergence, consistent with the inferred age of the pitviper recent stratum being younger than the divergence time between the garter snake and pitvipers (61.7 Ma; timetree.org; Kumar et al. 2017).

### The W Chromosome is GC-Rich and Repeat-Rich

**Fig. 3.**
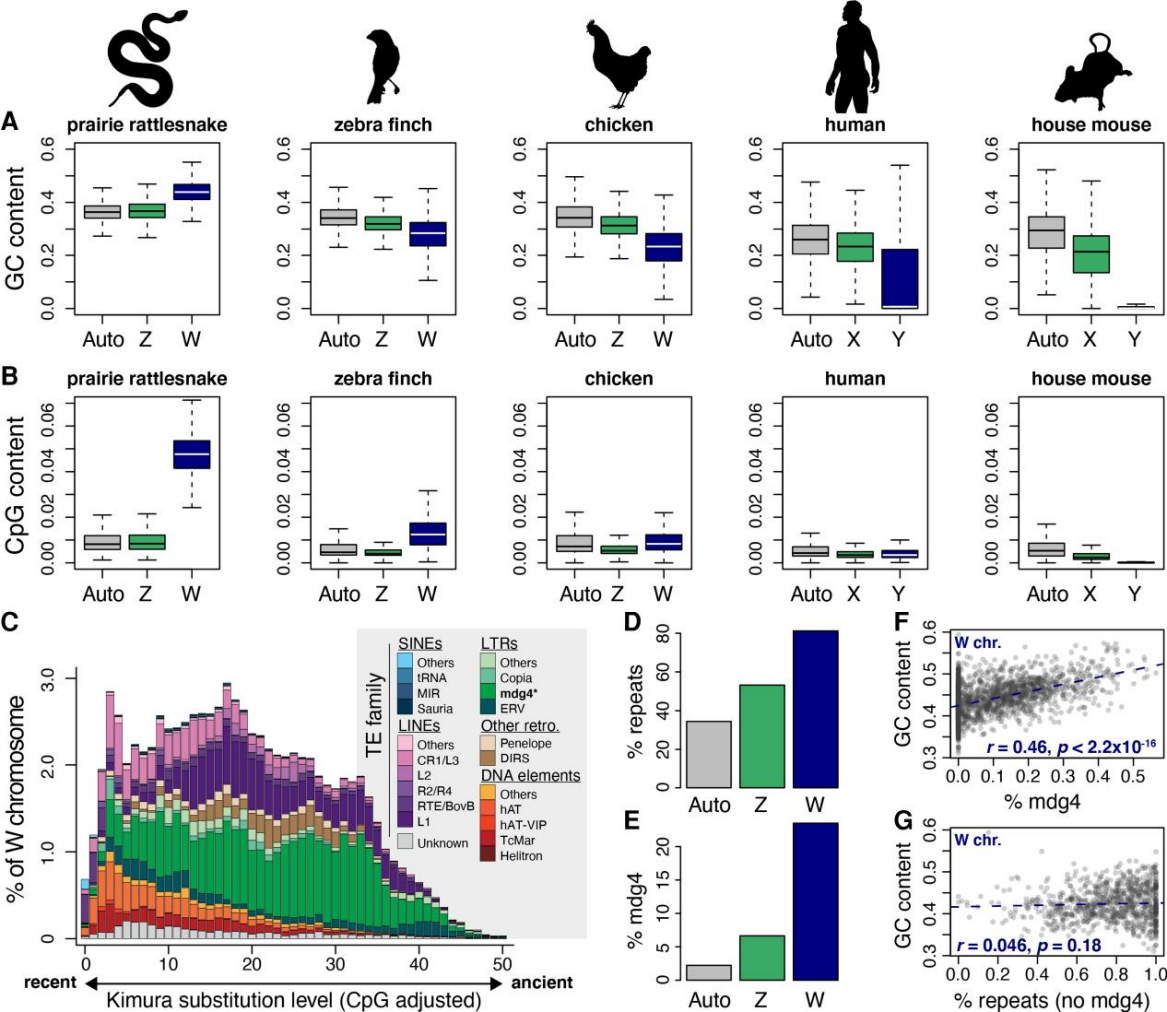
Composition of the W chromosome and comparison with other amniotes. (*A*) Distributions of GC content on autosomes, Z(X), and W(Y) chromosomes of the prairie rattlesnake and bird and mammal species. (*B*) Distributions of CpG content on autosomes, Z(X), and W(Y) chromosomes. (*C*) Age distribution of TEs on the W chromosome. Shaded bars represent the proportions of TE families with increasing age (i.e., % Kimura substitution level). Mdg4 elements are denoted with a “*”. (*D*) Proportions of autosomes, Z, and W chromosomes annotated as repeats. (*E*) Proportions of autosomes, Z, and W chromosomes annotated as mdg4 LTR elements. (*F*) Positive relationship between percent mdg4 elements and GC content across W chromosome scaffolds. (*G*) No correlation between percent total repeats (excluding mdg4 elements) and GC content across W chromosome scaffolds.

Our comparative analyses indicate that the rattlesnake W chromosome has a higher GC content (mean GC = 43.9%± 0.041%) than the Z chromosome (36.8% ± 0.041%) and autosomes (36.3% ± 0.04%; Mann–Whitney *U* tests, *P*-values <2.2 × 10^−16^; fig. 3*A*). The W chromosome also has a higher proportion of CpG sites than both the Z chromosome and autosomes (W = 4.8% ± 0.9%, Z = 0.97% ± 0.51%, autosomes = 0.96% ± 0.54%; Mann–Whitney *U* tests, *P*-values <2.2 × 10^−16^; fig. 3*B*). These results are surprising because the W chromosome is non-recombining, meaning that an accumulation of GC content though GC-biased gene conversion is not a plausible explanation for the higher overall GC content on the W chromosome relative to recombining chromosomes. To explore this result in the context of other amniotes with degenerated sex-limited chromosomes, we measured GC and CpG content in chicken, zebra finch, human, and mouse. In these comparisons, GC content on sex-limited W and Y chromosomes is significantly lower than autosomes and Z(X) chromosomes (Supplementary Table 4; Mann–Whitney *U* tests; *P*-values <0.05), the opposite pattern observed in prairie rattlesnake (fig. 3*A*). Similarly, CG dinucleotide (i.e., CpG) content is lower on the W and Y chromosomes of the bird and mammal species with the exception of the zebra finch (Mann–Whitney *U* tests, *P*-values <2.2 × 10^−16^; fig. 3*B*), which has slightly higher W-linked CpG content. Compared with the other species, CpG content on the rattlesnake W chromosome stands out as being nearly 5-fold higher than the Z chromosome and autosomes (fig. 3*B*).

The rattlesnake W chromosome also contains very high proportions of repeat element-derived sequences (fig. 3*C*), consisting of 81.15% total repetitive content (>2-fold higher than rattlesnake autosomes; fig. 3*D*) derived from a diversity of TEs. Such high TE density in the rattlesnake W is similar to sex-limited chromosomes of other species (Bachtrog 2005; Graves 2006; Smeds et al. 2015; Peona et al. 2021) and is consistent with the prediction that there is less efficient selection on the sex-limited chromosome to purge deleterious mutations due to the absence of recombination and lower effective population size than other chromosomes (Charlesworth and Charlesworth 2000). This finding strongly contrasts with the 15.39% repeat content reported in Singchat et al. (2020) based on the previously reported Indian cobra (*Naja naja*) W chromosome scaffold (Super-Scaffold_1000010; Suryamohan et al. 2020). However, we show that this scaffold was incorrectly identified as the W chromosome, and instead represents an autosomal chromosome scaffold (see Discussion; Supplementary Appendix; supplementary fig. S2A–B, Supplementary Material online). Furthermore, using the same approaches we used to identify candidate W-linked scaffolds in the rattlesnake, we identified a revised (and distinct) set of putative W chromosome scaffolds in the female Indian cobra assembly (supplementary fig. S2C, Supplementary Material online; Supplementary Data) totaling 35.9 Mb in length that do not include Super-Scaffold_1000010.

The most abundant TE family on the rattlesnake W chromosome are mdg4 (formerly known as “gypsy”) retroviral-like long terminal repeat (LTR) retrotransposons, which constitute 23.5% of our assembled W chromosome sequence (fig. 3*E*; supplementary table S5, Supplementary Material online). Autosomes and the Z chromosome contain much lower proportions of mdg4 elements overall (2.23% and 6.64%, respectively), with lower mdg4 retroelement densities across autosomal and Z-linked regions (i.e., 10 kb sliding windows) compared with the W chromosome (Mann–Whitney *U* tests, *P* < 2.2 × 10^−16^). Several potential mechanisms could explain this pattern. One possibility is that mdg4 elements on ancestral proto-sex chromosomes have persisted (i.e., experienced reduced decay) on the W chromosome following recombination suppression; a large proportion of relatively ancient mdg4 elements on the W chromosome would support this hypothesis. Alternatively, this pattern could be driven by an accumulation of mdg4 elements on the W chromosome independent of their evolution on the Z chromosome. The age distribution of mdg4 elements on the W chromosome (i.e., CpG-corrected Kimura distance) indicates prolonged activity, including a large proportion of recently active mdg4 elements (fig. 3*C*). W-linked mdg4 retroelements also tend to be longer in length than those on the Z chromosome and autosomes (mean length = 625.56 bp on the W chromosome versus 475.8 bp on autosomes and the Z chromosome; supplementary fig. S3, Supplementary Material online; Mann–Whitney *U* tests, *P*-values <4 × 10^−11^). It is also notable that in our analysis of full-length retroelements (below) we identify 327 full-length and potentially transpositionally-active mdg4 elements (i.e., with paired LTRs and open reading frames with intact *gag* and *pol* genes) on the W chromosome. The relative abundance of W-linked mdg4 elements potentially capable of self-replication argues for their accumulation after recombination suppression between the sex chromosomes.

### mdg4 Retroelements Drive GC-Richness on the W Chromosome

Given the high frequency of W-linked mdg4 retroelements, we asked if these elements may substantially contribute to compositional patterns on the W chromosome, such as the observed GC-richness. Indeed, W-linked mdg4 retroelement regions are GC-rich (mean GC = 47.4% ± 5.1% SD), significantly more so than L1 LINEs and CR1/L3 LINEs which are otherwise fairly abundant TE families in the entire rattlesnake genome and on the W chromosome specifically (34% ± 5.6% and 44.8% ± 7.8% GC, respectively; Mann–Whitney *U* tests, *P*-values <2.2 × 10^−16^; supplementary table S5, Supplementary Material online). Mdg4 elements are also significantly more GC-rich than the total background of non-mdg4 TEs (mean GC = 43.4% ± 12.8%; *P* < 2.2 × 10^−16^). We therefore tested whether regional GC content is associated with mdg4 element density, and find that GC content and mdg4 density are positively correlated across the W chromosome (Spearman’s rank correlation coefficient *r* = 0.46, *P* < 2.2 × 10^−16^; fig. 3*F*). This association persists when controlling for CpG content and total repeat content (Spearman’s partial correlation coefficient *r* = 0.15, *P* = 1.55 × 10^−11^). Furthermore, we find no correlation between GC content and total repeat content when mdg4 elements are removed (*r* = 0.046, *P* = 0.18; fig. 3*G*). These results support a view that the W chromosome has evolved higher GC content than autosomes and the Z chromosome primarily due to the proliferation of GC-rich LTR retroelements.

### The W Chromosome is a Refugium for Retroelements

**Fig. 4.**
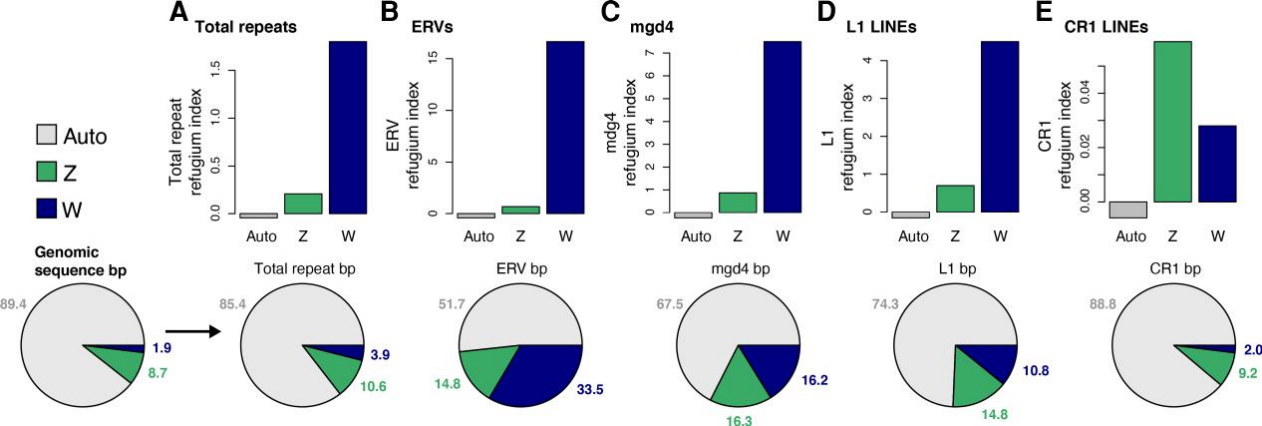
Refugium indexes for the autosomes, Z, and W chromosomes for total repeats (*A*) and ERV (*B*), mdg4 (*C*), L1 LINE (*D*), and CR1 LINE (*E*) retroelements, calculated based on observed an expected frequencies of repeat bp on the autosomes and sex chromosomes, assuming a uniform distribution. Bar plots in the top of each panel show calculated refugium index values; refugium index >0 indicates an excess of repeats; refugium index <0 indicates depletion of repeats relative to a uniform distribution. All autosomal refugium indices are negative. Pie charts at the bottom of each panel show the proportions of autosomal, Z, and W chromosome bp in the analyzed genome assembly compared with proportions of bp for each repeat element category.

We tested the hypothesis that the rattlesnake W chromosome acts as a refugium for retroviral-like elements by examining the frequencies of TE families, including full-length LTRs, across the genome and testing whether these fit a uniform distribution or if the W chromosome is exceptional with regard to genome-wide TE distributions. We used a *χ*^2^ framework (Peona et al. 2021) to compare observed and expected TE frequencies and to calculate a refugium index (RI) for autosomes, the Z, and the W, where positive RI values indicate an excess of elements compared with a uniform distribution. Indeed, we find that the W chromosome contains significant excesses of total repeat elements (RI = 1.8), ERVs (RI = 16.64), mdg4 (RI = 7.5), and L1 LINE elements (RI = 4.7; fig. 4*A–D*; table 2; supplementary table S6, Supplementary Material online). The observed distributions of TEs in each of these categories deviated significantly from a uniform expectation (total repeat *χ*^2^ = 14,226,221, *df* = 2, *P* < 2.2 × 10^−16^; ERV *χ*^2^ = 8,724,789, *df* = 2, *P* < 2.2 × 10^−16^; mdg4 *χ*^2^ = 17,352,014, *df* = 2, *P* < 2.2 × 10^−16^; L1 LINE *χ*^2^ = 6,583,173, *df* = 2, *P* < 2.2 × 10^−16^). The Z chromosome also has RI values consistent with an excess of TEs, yet these values are lower than those observed on the W (fig. 4*A–D*), with the exception of CR1 LINEs, for which the Z chromosome has a higher RI (0.059) than the W chromosome (0.028); observed frequencies of CR1 LINEs also deviate significantly from the uniform expectation (*χ*^2^ = 14,663, *df* = 2, *P* < 2.2 × 10^−16^). In contrast to patterns on the sex chromosomes, autosomes do not show evidence of excess TEs (i.e., RI <0; table 2).

**Table 2:** Refugium Index for Transposable Element Classes on the Autosomes, Z Chromosome, and W Chromosome

Element	Autosome RI	Z chromosome RI	W chromosome RI	χ^2^
Total repeats	–4.375	21.439	107.815	14,226,220***
ERVs	–42.164	69.973	1664.497	8,724,788***
mdg4	–24.472	87.749	750.403	17,352,014***
L1 LINEs	–16.849	70.555	470.236	6,583,173***
CR1 LINEs	–0.6362	5.9233	2.842	14,663***
fl-LTRs	–4.09	28.895	60.402	1,604,306***

χ^2^ tests were used to test for deviations from uniform expectation.

***P-value <2.2 × 10^−16^.

ERVs, endogenous retroviruses; fl-LTRs, full-length LTR retrotransposons.

We identified a total of 15,961 genome-wide full-length LTR retrotransposons (fl-LTRs), defined as having intact LTR and protein domains (see Methods), that were non-randomly distributed across the genome (*χ*^2^ = 384.21, *df* = 2, *P* < 2.2 × 10^−16^), with significant enrichment on the sex chromosomes. Of these, 572 (3.6%) are located on the W chromosome, corresponding to an RI of 0.89. The Z chromosome contains 1,786 fl-LTRs (11.2%; RI = 0.29). Autosomes contain the remaining 13,603 fl-LTRs (85%), significantly fewer than expected under a uniform expectation and corresponding to an RI of –0.047. In addition to the W chromosome having the highest fl-LTR RI, these full length retroelements constitute 13.2% of total W-linked sequence. These results, along with evidence of high RI for specific TE families, indicate that the rattlesnake W chromosome acts as a refugium for potentially transpositionally-active LTR retroelements. We further tested for evidence of sex differences in LTR-derived “toxicity” by calculating a toxicity index (Peona et al. 2021) based on identified fl-LTRs on autosomes and the sex chromosomes (see Methods). This analysis suggests no sex bias in toxicity, as the calculated index is very near zero (toxicity index = –0.03).

### W-Linked Gene Expression and Female-Specific Function

**Fig. 5.**
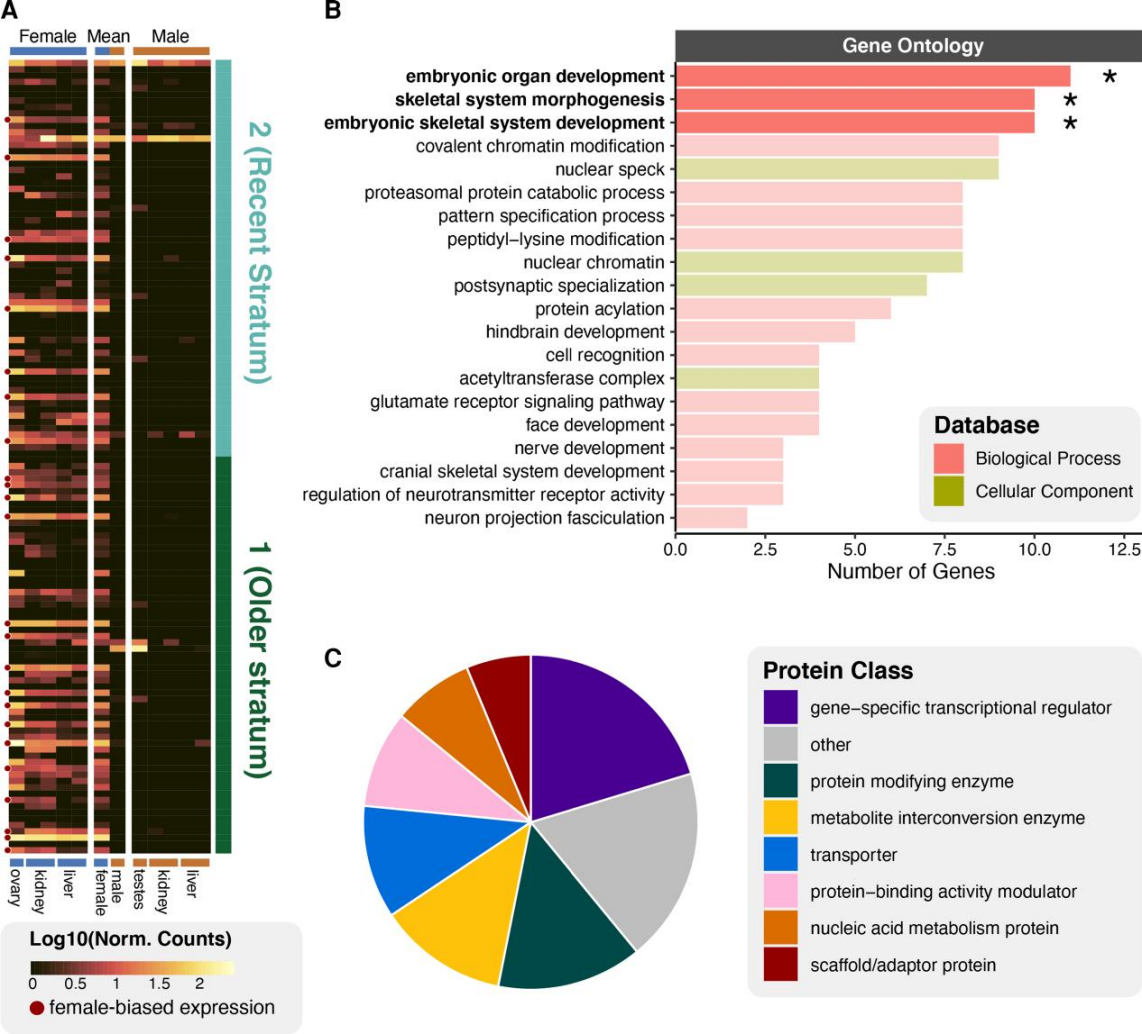
Gene expression and functional characterization of genes on the W chromosome. (*A*) Log_10_-normalized TPM expression counts for ovary, kidney, and liver in females are compared with testes, kidney, and liver in males. Female and male average expression values are displayed in the center. Bars at the right denote genes within the recent and older evolutionary strata. Genes with female-biased expression are denoted with red dots to the left of the heatmap. (*B*) Gene ontology categories (biological processes and cellular components) that comprise W-linked genes. Terms denoted with asterisks are significantly enriched for W-linked genes after FDR correction. (*C*) Panther protein classification for W-linked genes.

Gene expression analyses using female and male RNAseq data identified evidence of expression for 145 (66.2%) of the 219 annotated genes on the W chromosome (supplementary table S7, Supplementary Material online). Of these, 137 W gametologs (62%) show evidence of being expressed in female tissues (mean female TPM >0), and 31 (14.2%) have female-biased expression based on formal tests of “upregulation” compared with males (IHW *P*-values <0.05; fig. 5*A*; supplementary fig. S4, Supplementary Material online). This analysis revealed only two other female-biased genes on other chromosomes, indicating disproportionate W-linkage of genes with female-biased expression. Additionally, a total of 103 genes show evidence of female expression (raw expression count >0 in at least one female tissue) and no evidence of male expression, further emphasizing W-linkage and divergence between Z and W gametologs for these genes. Identified 1:1 ZW gametologs with evidence of female expression are similar in abundance in both the recent and older evolutionary strata (45 and 44 genes, respectively), although the older strata contain a higher proportion of genes with female-biased expression (25.4%) than the recent stratum (12.7%). Overall, evidence of W-linked gene expression indicates a degree of retained gene function across both recent and older evolutionary strata.

We tested whether genes on the W chromosome show any relevant links to female-specific traits and biological processes by characterizing functional annotations for W-linked genes using gene ontology (GO) term, pathway, and protein class classifications. Multiple enriched GO terms (i.e., FDR-corrected *P*-value <0.05) are directly relevant to embryogenesis, including embryonic organ development, skeletal system morphogenesis, and embryonic skeletal system development (fig. 5*B*). Other non-enriched terms represented by multiple W-linked genes relate to developmental processes, including facial development, cranial skeletal system development, hindbrain development, and nerve development. W-linked genes are not significantly enriched for any specific pathways, however several pathways relevant to embryo development involve genes on the W chromosome, including: activation of HOX genes during differentiation, activation of anterior HOX genes in hindbrain development during early embryogenesis, and programmed cell death (supplementary fig. S5, Supplementary Material online). Other functional categories involving W-linked genes relate to chromatin regulation (fig. 5*B*), including: covalent chromatin modification (biological process; nine genes), nuclear chromatin (cellular component; eight genes), acetyltransferase complex (cellular component; four genes), and the histone acetyltransferases acetylate histones pathway (four genes). Protein classifications indicate that the W chromosome also contains genes that function as gene-specific transcriptional regulators and protein modifying enzymes (fig. 5*C*). One of these genes, *LMTK3*, is a positive regulator of the estrogen receptor *ESR1* (Giamas et al. 2011), which plays a putative role in dosage compensation on the Z chromosome in rattlesnakes (Schield et al. 2019) and female-biased gene expression in other reptiles (Rice et al. 2017).

### Evolution of W-Linked Genes: Survival, Duplication, Selection, and Gene Conversion

A large percentage of W-linked genes (49.6%) occur in the recent stratum, representing a significant enrichment over the proportion of Z-linked genes in this region (19.7%; Fisher’s exact test, *P* = 2 × 10^−7^). In contrast, the older stratum of the W chromosome is depleted of genes relative to the Z chromosome (50.4% versus 80.3%, respectively; *P* = 0.0037). This suggests a greater degree of gene persistence/retention (or a slower decay rate) in the recent stratum of the W chromosome, though this pattern could be a simple consequence of more ancient recombination suppression in older strata. To account for this, we calculated the rate of W-linked gene decay in each stratum based on the number of ancestral genes lost on the W chromosome and estimated divergence times. Here, we calculate a W-linked recent stratum decay rate of 5 × 10^−6^ (i.e., five genes lost per million years), >2-fold slower than the W-linked older stratum rate (1.2 × 10^−5^), and consistent with a greater degree of gene retention in the recent stratum.

Translocation from autosomes is another mechanism to combat gene decay on the W chromosome (Mank 2012), in which autosomal genes are transferred to the W chromosome after recombination suppression between the sex chromosomes. We tested for evidence of translocated genes by identifying W-linked genes that lack a matched Z gametolog and are also not orthologous to genes on *Anolis* chromosome 6 (which shares common ancestry with caenophidian snake ZW sex chromosomes). We find 17 genes (7.7% of W-linked genes) with evidence of translocation to the W from autosomes (supplementary table S8, Supplementary Material online), indicating that both ancestral gene survival and gene gains from autosomes contribute to gene content on the W chromosome. Functional annotation of translocated genes do not show statistical enrichment for specific GO terms or pathways (supplementary fig. S6A–B, Supplementary Material online), however several genes have known roles in immune function (*H2Q9* and *RBM14*; see below) and the gene *VWA5A* may function as a tumor suppressor in humans (Martin et al. 2003). Other candidate autosome-W-translocated genes are uncharacterized (supplementary table S8, Supplementary Material online).

**Fig. 6.**
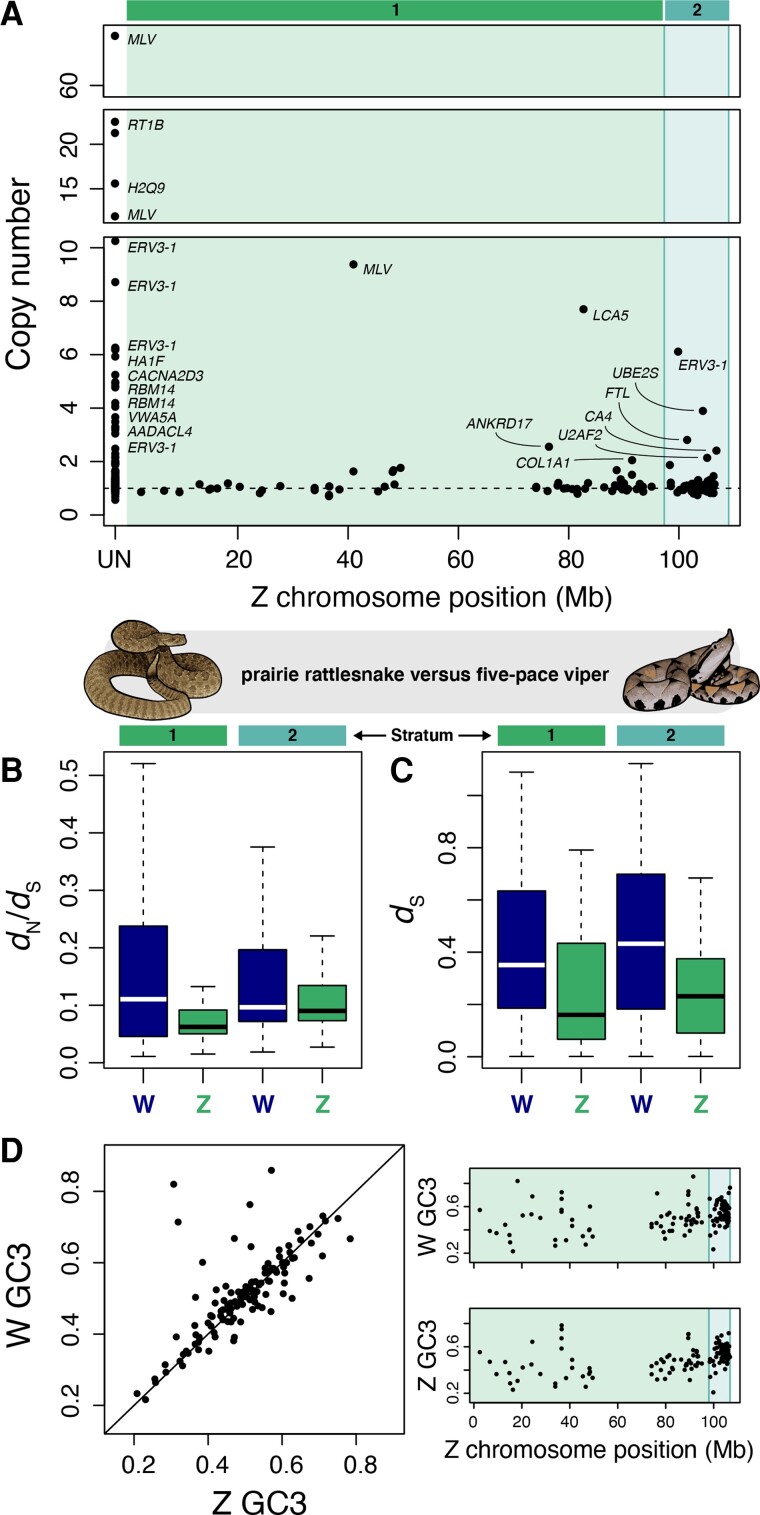
W-specific gene duplications, sex-linked divergence between pitviper species, and GC content at third codon positions (GC3) in rattlesnake ZW gametologs. (*A*) Estimated copy number for W-linked genes derived from comparisons with autosomal gene coverage, with points placed according to their position on the Z chromosome. The shaded regions correspond to strata 1 (older) and 2 (recent). Note that the *y*-axis is not continuous to display genes with high estimated copy number. Points for the genes with evidence of two or more copies are labeled. Gene names appearing more than once had multiple annotated positions on W-linked scaffolds. Genes without an identified 1:1 Z gametolog are labeled “UN” on the *x*-axis. (*B*) Distributions of *d*_N_/*d*_S_ between prairie rattlesnake and five-pace viper Z and W orthologs in stratum 1 and 2. (*C*) Distributions *d*_S_ between prairie rattlesnake Z and W orthologs. (*D*) GC3 content of ZW gametologs in prairie rattlesnake. The scatterplot to the left shows the relationship between GC3 for Z and W gametologs. The panels to the right show GC3 content for W and Z gametologs, respectively, based on Z chromosome position.

We further tested for evidence of W-specific gene duplications (i.e., ampliconic genes; Bachtrog 2013) in the rattlesnake by comparing sequencing depths between autosomes and the W chromosome, with the expectation that single-copy W gene depth would be half of that observed for autosomal genes. We infer that 21% of genes on the W chromosome are present in two or more copies, with a subset showing evidence of even greater degrees of amplification (fig. 6*B*; supplementary table S9, Supplementary Material online). Several of these genes lack apparent Z gametologs and are annotated as *MLV*-related proviral *ENV* polyprotein, *ERV3-1*, and *ERV-2*, which are present in at least 94, 47, and 6 total copies, respectively. Amplification of these genes is logical given that they are derived from retroviral-like elements (Cohen et al. 1985; Evans et al. 2003), which are abundant and active on the W chromosome (fig. 4). Other ampliconic genes include *RBM14*, which is involved in the activation of the innate immune response in humans (Morchikh et al. 2017), and multiple members of the vertebrate histocompatibility complex (*H2Q9*, *RT1B*, and *HA1F*). We infer that *H2Q9* and *RBM14* were translocated from autosomes to the W chromosome, with evidence of W-specific duplications indicating subsequent amplification following translocation. While these results suggest that gene duplication may have produced an expanded immune gene complement in females, we note that our approach cannot confirm whether these duplicates are functional because evidence for the multi-copy nature of these genes is based only on relative read depths rather than multiple reconstructed gene models in our annotation (likely due to their repetitive and structural complexity). Inferred duplicated genes were not significantly enriched for specific GO terms or pathways (supplementary fig. S6C–D, Supplementary Material online), though several genes are involved in response to estradiol, tissue and neuronal development, and skeletal morphogenesis.

Because it does not experience sexual recombination and has a low effective population size, we predict that the effects of linked selection and genetic drift hinder the efficiency of selection on the W chromosome to a greater extent than the rest of the genome. Comparing rates of between-species sequence divergence for the sex chromosomes provides a means to test whether these factors have indeed differentially influenced sequence evolution on the Z and W chromosomes since recombination suppression. We calculated the ratio of nonsynonymous to synonymous divergence (*d*_N_/*d*_S_) between prairie rattlesnake and five-pace viper for Z and W gametologs, and compared the distributions of *d*_N_/*d*_S_ with the expectation that higher values are indicative of less efficient purifying selection (Charlesworth and Charlesworth 2000). Importantly, we restricted these analyses only to genes for which we could identify reciprocal best hit orthologs to avoid comparisons between gene duplicates (see Methods). The W chromosome has significantly higher *d*_N_/*d*_S_ (0.44 ± 0.29) than the Z chromosome (0.26 ± 0.22; Mann–Whitney *U* test, *P*-value = 2.8 × 10^−6^; fig. 6*B*; supplementary table S3, Supplementary Material online), consistent with reduced efficacy of purifying selection on the W chromosome. This result is consistent across evolutionary strata, with higher W-linked *d*_N_/*d*_S_ in stratum 1 (i.e., older strata; W *d*_N_/*d*_S_ = 0.41 ± 0.29, Z *d*_N_/*d*_S_ = 0.26 ± 0.26; *P*-value = 0.0034) and stratum 2 (recent stratum; W *d*_N_/*d*_S_ = 0.46 ± 0.3, Z *d*_N_/*d*_S_ = 0.26 ± 0.19; *P*-value = 0.0003).

In addition to differences in effective population size between the sex chromosomes and the effects of selective interference on the W chromosome, it experiences a female-specific mutation rate because it is unique to the female germline. Investigations comparing rates of divergence between autosomes and the Z chromosome in snakes have demonstrated male-biased mutation rates (Vicoso et al. 2013; Schield et al. 2021), similar to patterns observed in birds (e.g., Axelsson et al. 2004; Smeds et al. 2015). We therefore expected to find higher mutation rates on the Z chromosome than W chromosome. Surprisingly, we find higher *d*_S_ between the prairie rattlesnake and five-pace viper W chromosomes (0.18 ± 0.21) than between the Z chromosomes (0.1 ± 0.09; *P*-value 0.02; fig. 6*C*; supplementary table S3, Supplementary Material online).

Faster divergence on the W chromosome could be driven by a higher female-specific mutation rate than previously estimated, although a more likely mechanism is intrachromosomal gene conversion, which has been observed among sex-limited chromosomes of diverse taxa (Slattery et al. 2000; Rozen et al. 2003; Backström et al. 2005; Wright et al. 2014; Chang and Larracuente 2019). A uniting feature in these examples is the presence of gene duplications, enabling gene conversion between paralogs on the sex-limited chromosome (Connallon and Clark 2010). The abundance of amplified gene copies on the rattlesnake W chromosome may present a similar repetitive substrate for intrachromosomal gene conversion. To test for gene conversion on the W chromosome, we compared the proportion of GC bases at third codon positions (GC3) between ZW gametologs, following Smeds et al. (2015). Here, the expectation under the GC-biased gene conversion model (Galtier et al. 2001) is that W-linked genes will have lower GC3 than their Z-linked counterparts in the absence of gene conversion on the W chromosome. However, the GC3 distribution for W gametologs (51.2% ± 12%) is not significantly lower than Z gametologs (50% ± 11%; Welch’s two-sample *t*-test, *P*-value = 0.27; fig. 6*D*; supplementary table S3, Supplementary Material online), and 56% of W genes have higher GC3 than the matching Z gametolog. This finding contrasts with the avian W chromosome, for example, which contains few ampliconic genes with higher GC3 than Z gametologs (e.g., *HINTW*; Ceplitis and Ellegren 2004; Backström et al. 2005; Smeds et al. 2015; Rogers et al. 2021), and suggests that intrachromosomal gene conversion may be comparatively frequent on the snake W chromosome, playing a role in shaping patterns of W-linked synonymous sequence divergence and GC content.

## Discussion

### Evolution of the Distinctive Rattlesnake W Chromosome

The rattlesnake W chromosome possesses a number of distinctive structural and evolutionary features that provide an expanded perspective of how non-recombining sex chromosomes evolve in vertebrates, illustrating a broader diversity of evolutionary trajectories for sex-limited chromosome evolution in comparison to established models from mammals and birds. We infer that recombination suppression between the sex chromosomes occurred in at least two steps, with the initial formation of an ancient evolutionary stratum (or strata) roughly 87 Ma followed by the formation of the recent stratum 40 Ma (fig. 1). The presence of at least two evolutionary strata is consistent with several previous investigations (Vicoso et al. 2013; Yin et al. 2016; Schield et al. 2019), although these previous studies, which lacked the resolution of the present W chromosome assembly, were unable to determine whether the recent stratum represents a unique recombination suppression event in pitvipers, or if this event took place earlier in colubriform or even caenophidian snake evolution (i.e., caenophidans possess homologous ZW sex chromosomes; Rovatsos et al. 2018). Based on comparative analyses of colubriform species and divergence between ZW gametologs, we infer that recombination suppression in the recent stratum occurred after the split between pitvipers and the garter snake (figs. 1–2), suggesting that restricted recombination evolved independently in the homologous region of the sex chromosomes of other lineages (e.g., colubrids and elapids). Whether recombination suppression in the recent stratum occurred prior to the common ancestor of Viperidae is unclear. Our divergence time estimate is very close to the estimated split between true vipers and pitvipers (42.8 Ma; Zheng and Wiens 2016), and additional analysis of sex chromosomes in viperine species will be necessary to confirm whether the recent stratum is a shared feature of the Viperidae.

The majority of the caenophidian sex chromosome pair, however, experienced more ancient recombination suppression (figs. 1–2). While we suspect that this region may encompass multiple evolutionary strata, recombination suppression likely occurred too closely in time to distinguish between independent events (fig. 1*C–D*). The inferred age range of the older strata is coincident with the ancestral split between Caenophidia (including Colubriformes and Acrochordoidea) and Henophidia (including boas and pythons) roughly 91 Ma (Kumar et al. 2017). This is particularly interesting because henophidian snakes have evolved independent XY and ZW sex chromosomes multiple times (Gamble et al. 2017; Augstenová et al. 2018), while all caenophidian snakes sampled to date have homologous ZW sex chromosomes (Matsubara et al. 2006; Rovatsos et al. 2015, 2018). The age of the older strata and ubiquitous presence of ZW sex chromosomes together indicate that the formation of ZW sex chromosomes was a major transition in early caenophidian evolution.

The rattlesnake W chromosome has evolved a unique complement of features since the establishment of recombination suppression. A particularly striking feature is relatively extreme GC-richness, which contrasts sharply with sex-limited chromosomes of other amniote species (fig. 3). Chromosomes lacking sexual recombination are expected to have a lower frequency of GC nucleotides than recombining genomic regions because GC-biased gene conversion is expected to be less prevalent or absent altogether (Eyre-Walker and Hurst 2001; Galtier et al. 2001; Duret and Galtier 2009). Indeed, comparisons reveal a pattern of low GC content on the sex-limited chromosome across other ZW and XY amniote vertebrates (fig. 3). We find that GC-richness on the rattlesnake W chromosome coincides with an abundance of GC-rich mdg4 elements, which comprise over 23% of the rattlesnake W chromosome (figs. 3–4; supplementary table S5, Supplementary Material online). The relative abundance of full-length mdg4 elements on the W chromosome further suggests that mdg4-driven GC-richness has evolved since the onset of recombination suppression between the sex chromosomes, which promoted the retention of these potentially transpositionally-active elements.

Our finding that mdg4 element insertion has played a dominant role in the evolution and divergence of the rattlesnake W chromosome raises questions about what features of this particular element may have favored its invasion of the W, and what the biological ramifications of this invasion may be. Studies in *Drosophila* have indicated that mdg4 elements may accumulate in specific genomic regions through a feed–forward process in which the presence of an existing element significantly increases the probability of insertion of additional new elements into adjacent genomic sequences (Labrador et al. 2008). Evidence that the rattlesnake W chromosome is a major hotspot for mdg4 insertion is consistent with this model, which may partially explain why mdg4 elements have accumulated at such high frequencies. Mdg4 elements are known to be transmitted as retrovirus-like pro-viral particles (Labrador et al. 2008), which suggests they are highly capable of horizontal transfer. Unlike most LTR- and retroviral-like elements that target euchromatic open chromatin (Scherdin et al. 1990; Schröder et al. 2002; Wu et al. 2003), mdg4 elements tend to be site-specific and target heterochromatic regions (Labrador et al. 2008). In *Drosophila*, mdg4 elements are also transmitted through female oocytes, and insert in germline tissue via a piRNA-dependent mechanism, suggesting their potential to preferentially target the heterochromatic W chromosome and female germline tissues (Dej et al. 1998). These elements are also known to modify chromatin and to form insulator sequences (i.e., elements with enhancer-blocking activity; Labrador et al. 2008). It is thus plausible that mdg4 elements could have contributed to the suppression of recombination between regions of the Z and W chromosomes and associated divergence of Z versus W gametolog expression via insertion of insulator sequences on the W chromosome. Future comparative studies that integrate data on gene expression, chromatin accessibility, and chromatin structure (e.g., ATACseq and Hi-C) would be valuable for testing these hypotheses regarding the potential functional ramifications of mdg4 element insertions on the W chromosome.

In addition to patterns of GC-richness driven by mdg4 retroelements, intrachromosomal gene conversion also appears to have contributed to the GC-richness of the rattlesnake W chromosome and may represent a mechanism to counteract W-linked gene decay. Using simulations, Marais et al. (2010) showed that gene conversion between Y-linked gene copies can be beneficial for opposing Muller’s Ratchet, with greater gene copy number providing an enhanced benefit. Other empirical examples also show a prevalence of gene conversion between amplified gene families on Y and W chromosomes (e.g., Rozen et al. 2003; Backström et al. 2005; Connallon and Clark 2010; Rogers et al. 2021)—the taxonomically widespread nature of gene conversion on sex-limited chromosomes indeed suggests that it may be advantageous in tempering rates of gene decay. Several lines of evidence support that the counter-degeneration effects of gene conversion are relevant to snake W chromosome evolution. Most generally, the observation of W genes with higher GC3 than matched Z gametologs (fig. 6; supplementary table S3, Supplementary Material online) suggests the presence of GC-biased gene conversion, analogous to the evolution of the *HINTW* gene family on the avian W chromosome (Ceplitis and Ellegren 2004; Backström et al. 2005; Nam and Ellegren 2008). Furthermore, we estimate that the recent stratum has experienced a slower rate of gene decay than older strata, combined with a greater number of W-specific gene duplications and higher *d*_S_ values on average (fig. 6). Based on this complement of patterns, we hypothesize that gene duplications have provided an evolutionary substrate for intrachromosomal gene conversion across the W chromosome, especially within the recent stratum. It is also possible that the mdg4-dominated repeat landscape of the W chromosome has further enhanced the propensity for intrachromosomal gene conversion.

### Retroelements and the Evolution of Sex-Linked Mutational Load

Among the most distinctive features of the rattlesnake W chromosome is that it is more than 80% repetitive, far surpassing the repeat content of autosomes and the Z chromosome (fig. 3; supplementary table S5, Supplementary Material online; Schield et al. 2019). In contrast, Singchat et al. (2020) reported much lower repeat content based on the previously published genome assembly for the Indian cobra (*N. naja*) that contained a scaffold identified as the W chromosome (Super-Scaffold_1000010; Suryamohan et al. 2020). The surprising contrast in repeat landscapes between the two species, along with other preliminary comparative analyses, led us to question the W-linkage of Indian cobra Super-Scaffold_1000010, and we performed a series of analyses designed to test whether the scaffold is instead autosomal (and thus misidentified as the W chromosome). Indeed, comparative mapping of male and female reads combined with synteny analyses indicate that this Indian cobra scaffold represents a fragment of chromosome 6 (Supplementary Appendix; supplementary fig. S2A-B, Supplementary Material online). Based on these results, we conclude that prior characterization of this presumed W chromosome in the Indian cobra was in error, and instead described characteristics of an autosome wrongly inferred to represent the W chromosome in that species (a list of candidate W-linked Indian cobra scaffolds identified in our study is provided in Supplementary Data). As such, the repeat-poor inference reported in Singchat et al. (2020) is not representative of the snake W chromosome repeat landscape, and our findings presented here for the rattlesnake W chromosome provide, to our knowledge, the first accurate characterization of the repeat element accumulation and composition on a snake W chromosome.

Accumulation of repeat content on the rattlesnake W chromosome is consistent with the large body of empirical evidence that TE activity and accumulation drives the evolution of highly repetitive sex-limited chromosomes (Bachtrog 2013; Smeds et al. 2015; Tomaszkiewicz et al. 2017; Peona et al. 2021). Here, TE insertions likely persist at a higher frequency on the W chromosome because selection is less efficient against slightly deleterious mutations due to the magnified effects of genetic drift and selective interference—ultimately a consequence of recombination arrest (Charlesworth et al. 1986; Charlesworth and Charlesworth 2000). While mechanisms (e.g., gene conversion) may exist to oppose genetic decay, at a broad scale their effects appear to be limited relative to the dominant impacts of retroelements, including full length elements capable of transpositional activity, in shaping the mutational landscape of the W chromosome. Moreover, the relevance of these factors supports the view that the W chromosome is the product of antagonistic evolutionary mechanisms, culminating in a fine balance between decay and retention of ancestral genomic regions and genes.

The repetitive nature of sex-limited chromosomes may have important implications for sex-biased mutational load and genome function though epistatic effects on chromatin structure. The observation that W(Y) chromosomes act as reservoirs for TEs that have large-scale effects on gene expression during aging due to breakdowns in heterochromatin has led to the “toxic Y” hypothesis (Brown et al. 2020b; Nguyen and Bachtrog 2020; Wei et al. 2020), and spurred the development of metrics to quantify the toxicity of sex-limited chromosomes and the degree to which they act as TE refugia (Peona et al. 2021). We find that the rattlesnake W chromosome is highly enriched for retroelements, including multiple retroviral-like elements (fig. 4; table 2; supplementary table S6, Supplementary Material online), which are similarly abundant on the W chromosome in bird species (Peona et al. 2021). These include a disproportionate number of full-length elements that have likely been protected from purifying selection due to selective interference on the W chromosome, providing the first evidence that the snake W chromosome acts as a refugium for self-replicating TEs. This suggests the W chromosome may be a source of higher mutational load in female snakes, with possible downstream impacts on gene regulation and genomic instability analogous to those reported for birds (Peona et al. 2021) and *Drosophila* (Nguyen and Bachtrog 2020; Wei et al. 2020). The potential for genetic incompatibilities to arise on the W chromosome may have additional downstream impacts relevant to speciation, as the build-up of mutational load could drive reproductive isolation between snake lineages, similar to that suggested in birds (Peona et al. 2021). Intriguingly, we do not find support for female-biased toxicity based on the toxicity index, as both Z and W chromosomes are enriched for full-length elements relative to autosomes, yielding an index near zero (see Results). An important caveat to consider, however, is that the fragmentary nature of our W chromosome assembly may bias our inference of full-length elements (i.e., it likely represents a lower-bound on the true number of full-length elements), compared with the contiguous assembly of the Z chromosome and autosomes (table 1; Schield et al. 2019). It therefore remains an open question if there is truly no sex bias in “toxicity”, and additional analysis of the presence and activity of full-length elements based on contiguous W chromosome assemblies will be useful for further testing hypotheses related to female-specific mutational load.

### Specialization of the W Chromosome for Female Function

Despite substantial degeneration of the rattlesnake W chromosome, it has retained a subset of genes that may be important for female-specific function (fig. 5). Indeed, our characterization of W-linked genes indicates retention of genes involved in reproduction, development, and transcriptional regulation. Specifically, the W chromosome is enriched for genes involved in embryonic development (i.e., skeletal and nervous system development; fig. 5), and also houses multiple genes involved in early developmental differentiation (e.g., HOX genes), programmed cell death, and chromatin regulation. One particular retained gene on the W chromosome, *LMTK3*, is notable because it functions as a positive regulator of the estrogen receptor *ESR1* (Giamas et al. 2011), which is known to play a role in promoting female-biased gene expression in vertebrates (Nilsson et al. 2001; Rice et al. 2017). A previous study found evidence for an evolutionary increase in the abundance of estrogen response elements (cis-regulatory elements bound by ESR1) on the rattlesnake Z chromosome that may facilitate gene- or region-specific dosage compensation (Schield et al. 2019). It is therefore plausible that survival of the *LMTK3* W gametolog was favored by selection because it is necessary for activation of *ESR1* related to its role in estrogen-driven upregulation of dose-sensitive genes in females.

Several genes involved in immune function have also translocated to the W chromosome and or experienced W-specific amplification through apparent gene duplication (e.g., *H2Q9*, *HA1F*, *RBM14*, and *RT1B*). Why translocation and pronounced duplication of such genes has evolved is unclear, but it is logical that an associated increase in immune function is beneficial to female survival and reproduction. In the case of W-specific duplications of immune genes, one possibility is that selection has favored amplification to compensate for lower expression on the highly heterochromatic W chromosome, similar to explanations for expanded gene families on the *D. miranda* neo-Y chromosome (Bachtrog 2006, 2020). An alternative explanation, analogous to the hypothesized role of *HINTW* expansion in birds (Backström et al. 2005), is that duplication is simply favorable as a mechanism to safeguard against genetic decay and ultimate loss of immune genes on the W chromosome.

### Conclusion

Snakes inspired foundational work that established prevailing views of sex chromosome evolution (Ohno 1967), yet characterizations of snake sex chromosome genomic structure and function have since lagged behind those of other vertebrate groups, leaving a major gap in our understanding of the forces that shape the evolution of sex chromosomes in vertebrates. Here, we provide the first detailed analysis of the W chromosome from a caenophidian snake, the prairie rattlesnake. Our findings reveal a unique complement of structural features on the W chromosome that bear similarities and contrasts with other species, providing an expanded view of vertebrate sex chromosome evolution. The diverse and often conflicting dynamics between specialization of the W chromosome for vital female-specific functions, retention of critical genes, retroelement proliferation, and ongoing genetic decay highlight the complexity of unique evolutionary forces that shape sex chromosomes. These findings also raise new questions for future comparative study, including the following: 1) how has recombination suppression proceeded in other caenophidian lineages, 2) has specialization for female function followed independent evolutionary trajectories in distinct snake ZW and XY systems, 3) how have mdg4 elements contributed to recombination suppression, chromatin structure, and divergence of gene expression and function on the W chromosome, and 4) how does female-specific mutational load and the potential “toxicity” of the W chromosome impact genomic regulation and are these effects relevant to speciation? Finally, though linked-read sequencing enabled the assembly of W chromosome scaffolds, these remain fragmentary likely due to high heterochromatin and repetitive content of the chromosome. Additional scaffolding using long-read technologies and Hi-C chromatin contact data (especially for trios of parents and offspring) holds promise for increasing the contiguity of highly-degenerated sex chromosome assemblies in the rattlesnake and other snake species.

## Materials and Methods

### Assembly of Prairie Rattlesnake W Chromosome Scaffolds

We used the prairie rattlesnake reference genome assembly and annotation (Schield et al. 2019; NCBI BioProject PRJNA413201) for analyses of autosomes and the Z chromosome. To generate sequence data for the W chromosome, we sampled a female prairie rattlesnake from the same population as the male genome animal. Liver tissue was snap-frozen in liquid nitrogen and stored at –80 °C. Genomic DNA was extracted and a 10x Genomics Chromium library was prepared to enable linked-read sequencing on an Illumina NovaSeq 6000 using 150 bp paired-end reads. We assessed sequence quality using FastQC (http://www.bioinformatics.babraham.ac.uk/projects/fastqc) and summarized the results using MultiQC (Ewels et al. 2016), which are shown in supplementary fig. S1, Supplementary Material online. We performed genome sub-assembly using the 10× Genomics Supernova assembler v2.1.1 (Weisenfeld et al. 2017) specifying 560 million input reads based on calculations of recommended fold genome-coverage and the estimated genome size. We used the “pseudohap2” option to generate scaffold sequences in order to retain as many potentially W-linked scaffolds as possible.

We identified W-linked scaffolds by first performing a homology search of all female scaffolds against the male genome assembly using MashMap (Jain et al. 2018), using a 95% identity threshold and the one-to-one filtering option. We removed high-similarity hits to autosomes and scaffolds that were less than 5 kb in length and retained hits to the Z chromosome and also any scaffolds that did not have significant sequence similarity to the male reference (i.e., female-specific scaffolds). This procedure is expected to enrich for a list of W-linked, Z-linked, and pseudoautosomal region (PAR) scaffolds from the female genome assembly. We then filtered scaffolds with hits to the 6.8 Mb PAR region, leaving 15,254 putatively sex-linked scaffolds.

We then used comparative mapping of whole genome resequencing reads from both sexes to distinguish W- and Z-linked female scaffolds. We quality trimmed reads (supplementary table S1, Supplementary Material online) using Trimmomatic v0.39 (Bolger et al. 2014) with the settings LEADING:20 TRAILING:20 MINLEN:32 AVGQUAL:30. We mapped filtered reads to all female scaffolds using default settings in BWA mem v0.7.17 (Li and Durbin 2009), then removed mapped reads with quality scores below Q30 using Samtools v1.1 (Li et al. 2009). We calculated sequencing depth per scaffold using Mosdepth (Pedersen and Quinlan 2018) with default parameters. We then used the distribution of read depths from scaffolds with best hits to autosomes to calculate median autosomal depth per sex.

Statistical methods used to identify W-linked sequence based on comparative mapping have different associated power and false-positive rates, with the potential to spuriously assign sex-linkage to autosomal regions or vice versa. To address this concern, we performed comparative mapping analyses in chicken (*Gallus gallus*) using available female and male resequencing data (Cortez et al. 2014; NCBI SRA accessions SRR958465 and SRR958466) mapped to version 6 of the *G. gallus* genome, which includes an assembled W chromosome (Hillier et al. 2004; Bellott et al. 2017). We filtered and mapped reads following the methods described for rattlesnake data and calculated mean read depth in genomic windows using Mosdepth. The mean length of female rattlesnake scaffolds was 10,013.8 bp, so we measured read depth in the chicken using 10 kb windows for comparison. We then counted the number of true and false positives when identifying putative W-linked chicken regions using two alternative approaches. First, we calculated

$$\mathrm{lo}g_{2}\left(\frac{\mathrm{female}\;\mathrm{autosome}-\mathrm{normalized}\;\mathrm{depth}}{\mathrm{male}\;\mathrm{autosome}-\mathrm{normalized}\;\mathrm{depth}}\right)$$

per genomic window, where autosome-normalized depth is equal to the mean read depth per window divided by the autosomal median read depth for each sex. We refer to this measure as “log_2_FM”. The expectation for autosomes is Log_2_FM = 0. Z-linked sequences, on the other hand, are expected to have Log_2_FM = –1 and W-linked sequences are expected to have Log_2_FM ∼2. To accommodate variance in read depths among genomic windows, we identified putative W-linked windows using a threshold of log­_2_FM ≥ 1. Second, we calculated the autosome-normalized proportion of female read depth per genomic window. Each window with a female read depth proportion greater than the third quartile threshold plus 1.5 times the inter-quartile range (i.e., Q3 + 1.5×IQR) was considered putatively W-linked. Each approach had high power to detect W-linked genomic windows (log_2_FM power = 0.954; Q3 + 1.5×IQR power = 0.97). Despite having slightly greater power, the Q3 + 1.5×IQR method also suffered nearly an order of magnitude higher false-positive rate (log_2_FM false-positive rate = 0.0049; Q3 + 1.5×IQR false-positive rate = 0.025). Based on the mapping experiment in chicken, we used the more conservative log_2_FM threshold to identify W-linked scaffolds in the prairie rattlesnake. We calculated log_2_FM per female scaffold, identified putative W-linked sequences using the Log_2_FM ≥ 1 threshold, then cross-referenced these with our list of putative sex-linked scaffolds from the initial homology procedure using the male reference genome. This yielded 2,139 female scaffolds with overlapping evidence of W-linkage (table 1).

### Annotation of the W Chromosome

We annotated repeat elements and protein coding genes to facilitate comparative analyses of sex-linked genes and to characterize the composition and structure of the W chromosome. Repeat elements were annotated using RepeatMasker v4.0.8 (Smit et al. 2015), leveraging a Bov-B/CR1 LINE homology database generated using multiple squamate genomes (Pasquesi et al. 2018), tetrapod elements included in RepBase release 20181026 (Bao et al. 2015), and known and unknown consensus elements from a snake-specific repeat library (Castoe et al. 2013; Pasquesi et al. 2018; Schield et al. 2019).

We used MAKER v2.31.10 (Cantarel et al. 2008) to produce an initial annotation of protein-coding genes in W-linked scaffolds using empirical evidence for gene prediction (settings est2genome = 1 and protein2genome = 1). Empirical evidence included the *de novo* transcriptome assembly used in the male prairie rattlesnake genome assembly (Schield et al. 2019) combined with a *de novo* transcript assembly derived from 18 tissues from female prairie rattlesnakes (supplementary table S2, Supplementary Material online). Tissue samples were collected and immediately snap frozen in liquid nitrogen and stored at –80 °C. Total RNA was extracted using Trizol and poly-A selected mRNA libraries were generated and sequenced on an Illumina HiSeq 2500 using 100 bp paired-end reads. Reads were randomly subsampled using the sample tool in seqtk v1.3-r106 (www.github.com/lh3/seqtk) to retain two million reads per sample. We then generated the transcriptome assembly using Trinity release 2014-07-17 (Grabherr et al. 2011) with the –trimmomatic flag to incorporate upfront quality trimming by Trimmomatic (Bolger et al. 2014) with default settings.

The *de novo* transcriptomes were supplied to the argument “est”, along with protein datasets for all annotated protein-coding genes of *Anolis carolinensis* (Alfoldi et al. 2011), *Python molurus bivittatus* (Castoe et al. 2013), *Thamnophis sirtalis* (Perry et al. 2018), *Ophiophagus Hannah* (Vonk et al. 2013), and *Deinagkistrodon acutus* (Yin et al. 2016) supplied to the argument “protein”. An initial round of MAKER was run with default settings, except that we specified max_dna_len = 300,000 and split_hit = 20,000. The resulting 386 gene models were used to optimize gene prediction parameters in Augustus v3.2.3 (Stanke and Morgenstern 2005), which we used for gene prediction in a second run of MAKER that was identical to the first, except for specifying est2genome = 0 and protein2genome = 0 and with Augustus parameters supplied to the “augustus_species” setting. The resulting annotation contained 213 protein-coding gene models (table 1).

We further used transcript data to improve the contiguity of the W chromosome assembly. We gathered the existing assembly together with gene models from MAKER in GFF3 format and mapped all female RNAseq read data used in the *de novo* transcriptome using default settings in BWA mem (Li and Durbin 2009). These data were then analyzed using AGOUTI v. 0.3.3-dirty (Zhang et al. 2016) with default settings except for minMQ = 20 and maxFracMM = 0.05. AGOUTI further scaffolded 206 of the 2,139 W chromosome scaffolds into 94 final scaffolds, resulting in a final assembly of 2,027 scaffolds with an N50 = 13,252 bp. We again performed repeat and gene annotations following the same procedures as above, resulting in 219 protein models after the second run of MAKER (table 1). We ascribed gene IDs based on homology for 134 of these gene models using a reciprocal best BLAST (Altschul et al. 1990; with an e-value threshold of 1 × 10^−5^) and stringent one-way BLAST (with an *e*-value threshold of 1 × 10^−8^) searches against protein sequences from NCBI for *Anolis*, *Python*, and *Thamnophis*.

### Sex Chromosome Homology and Identification of Evolutionary Strata

Previous investigations have hypothesized the presence of at least two evolutionary strata between Z and W chromosomes in caenophidian snakes (Vicoso et al. 2013; Yin et al. 2016; Schield et al. 2019). Comparisons of Z and W-linked gametologs in the context of the Z chromosome assembly for prairie rattlesnake provide the opportunity to examine the structure, age, and number of evolutionary strata in greater detail. To establish the structure of the assembled W chromosome in relation to the Z chromosome, we used MashMap (Jain et al. 2018) to anchor W-linked scaffolds with sufficient sequence similarity to homologous regions of the Z chromosome. We specified “-f one-to-one” to limit matches to reciprocal best hits, specified a minimum search length of 5 kb, and filtered hits with lower than 90% sequence similarity.

We identified ZW gametologs using tBLASTx (Altschul et al. 1990) searches between annotated coding sequences (CDS) with the setting “-max_hsps 1” and an e-value threshold of 1 × 10^–5^. We then extracted reciprocal best BLAST hits as 1:1 gametologs, yielding 125 1:1 gametolog pairs. We translated nucleotide sequences for each pair and aligned amino acid sequences using Clustal Omega (Sievers et al. 2011), then used PAL2NAL (Suyama et al. 2006) to generate codon-based nucleotide alignments. We estimated synonymous (*d*_S_) and nonsynonymous (*d*_N_) divergence between gametologs using the codon model in PAML (Yang 2007), and filtered pairs with *d*_S_ above 2 and below 0.001. To transform synonymous divergence estimates to divergence times, we calculated a lineage-specific mutation rate based on divergence between *Crotalus* and *Anolis* autosomal genes. We identified orthologous genes using a reciprocal best BLAST search, which yielded 11,277 1:1 autosomal orthologs. We aligned orthologs and calculated *d*­_S_ as described above, then obtained a mutation rate estimate by dividing median *d*_S_ (0.94) by 167 million years, the estimated divergence time for *Crotalus* and *Anolis* from TimeTree (Kumar et al. 2017), yielding a lineage-specific autosomal mutation rate of 2.8 × 10^−9^ per site per year. To account for sex-linked mutational biases, we multiplied this rate by the Z chromosome:autosome mutation rate ratio (*μ*_Ζ_/*μ*_A_) of 1.1 estimated in Schield et al. (2021). The W chromosome is present only in females and therefore has a female-specific mutation rate. While this rate is unknown in rattlesnakes, estimates of the male to female mutation rate ratio in birds, which have similar estimates of male-biased mutation rates to snakes (Vicoso et al. 2013; Schield et al. 2021), range from two to four (Nam and Ellegren 2008). To accommodate female-specific mutation on the W chromosome, we conservatively used a female mutation rate equal to one-fourth of the autosomal rate, adding this to the inferred rate for the Z chromosome to produce a combined sex-linked divergence rate (3.1 × 10^−9^ + 0.7 × 10^−9^ = 3.8 × 10^−9^). We transformed *d*_S_ estimates for ZW gametologs to time using this rate, then calculated the mean divergence time per stratum. For comparison, and to obtain a range of approximate divergence times per stratum, we repeated this procedure using a squamate mutation rate derived from 4-fold degenerate sites (2.4 × 10^−9^; Green et al. 2014), which, after conversion to the sex-linked divergence rate was equal to 3.24 × 10^−9^.

We investigated whether the recent stratum is present in non-pitviper colubroids by mapping female and male western terrestrial garter snake (*Thamnophis elegans*, a colubrid) read data (Vicoso et al. 2013; supplementary table S1, Supplementary Material online) to the prairie rattlesnake genome using bwa mem v0.1.17 (Li and Durbin 2009) after quality filtering reads using Trimmomatic v0.39 (Bolger et al. 2014) with settings described for other analyses above. We removed mapped reads with quality scores lower than Q30 using Samtools v1.1 (Li et al. 2009) and quantified read depths in 10 kb sliding windows using Mosdepth (Pedersen and Quinlan 2018). We calculated log_2_FM per window on the Z chromosome based on median autosomal read depths per sex, following our methods used for discovery of sex-linked genomic regions. We repeated these steps using resequencing data from three pitviper species: five-pace viper (*Deinagkistrodon acutus*; Yin et al. 2016), pygmy rattlesnake (*Sistrurus miliarius*; Vicoso et al. 2013), and prairie rattlesnake (supplementary table S1, Supplementary Material online). If recombination suppression in the recent stratum predates the common ancestor of the garter snake and pitvipers, log_2_FM would be intermediate between –1 and 0 in this region, corresponding to expectations for Z-linked and autosomal regions, respectively. Alternatively, log_2_FM values consistently near –1 would indicate that garter snake sex chromosomes experienced recombination suppression in this region independently of pitvipers.

### GC and Repeat Content on the W Chromosome

We measured GC content, CpG content, and repeat content on the W chromosome using custom Python scripts (https://github.com/drewschield/rattlesnake_w_chromosome). GC content was measured as the proportion of G and C nucleotides per scaffold after removal of ambiguous bases. Similarly, CpG content was measured as the proportion of CG dinucleotides, and repeat content was measured as the proportion of bases per scaffold annotated as repeats. We also measured GC and CpG content on the prairie rattlesnake Z chromosome and autosomes and across the genomes of chicken (*G. gallus* version GRCc6a; Hillier et al. 2004; Bellott et al. 2017), zebra finch (*Taeniopygia guttata* version bTaeGut2.pat.W.v2; Warren et al. 2010), human (*Homo sapiens* version GRCh38.p13; Venter et al. 2001) and house mouse (*Mus musculus* version GRCm39; Waterston et al. 2002) in 10 kb windows. The reference genomes for each of these species include representative assembled scaffolds for Z(X) and W(Y) sex chromosomes.

Measures of repeat content were based on the repeat annotation described above, and we used the CpG-corrected Kimura 2-parameter distance as a measure of the age distribution for specific element families. Several TE families were abundant on the W chromosome, including multiple LTR retrotransposons—mdg4, L1 LINEs, CR1/L3 LINEs, and ERVs (see Results). In addition to total repeat content, we measured the proportion of bases from these TE families per W-linked scaffold, and in 10 kb windows on the Z chromosome and autosomes. We compared GC and CpG content of mdg4, L1, and CR1/L3 elements to their densities on the autosomes, Z chromosome, and W chromosome, and examined the distributions of GC and CpG content for each of these families on the W chromosome.

### Analysis of Retroelements

We compared the frequencies of repeat elements on autosomes and the sex chromosomes to test the hypothesis that there is a uniform distribution of repeats across the genome. Following Peona et al. (2021), we first determined the expected repeat-derived bp by assuming that the total length of repeats is proportional to the total length of autosomes, Z, and W chromosomes after removing ambiguous bases. We then compared observed values to the expected values to calculate the RI for each chromosome class using the formula:

$$\mathrm{Refugium}\;\mathrm{Index}=\;\frac{\text{\%}TE_{obs}-\;\text{\%}TE_{exp}}{\text{\%}TE_{exp}}.$$

The RI provides information on whether the autosomes and sex chromosomes have a depletion (RI <0) or excess (RI >0) of repeat elements compared with a uniform distribution. We tested for significant deviation from a uniform distribution using *χ*^2^ tests. We repeated these analyses for specific abundant retroelements on the W chromosome (i.e., mdg4, ERVs, L1 LINEs, CR1/L3 elements).

To compare frequencies of LTR retroelements capable of self-replication across the genome, we identified full-length elements with intact LTR and protein domains (fl-LTRs) using LTRharvest (Ellinghaus et al. 2008) and LTRdigest (Steinbiss et al. 2009). We filtered results using Pfam (Mistry et al. 2021) and GyDB (Llorens et al. 2010) hidden Markov model profiles for LTR retrotransposon proteins. We then compared observed and expected numbers of fl-LTRs to calculate the RI for autosomes and the sex chromosomes.

To examine evidence of sex differences in toxicity on the basis of homogametic and heterogametic linkage of fl-LTRs, we calculated the toxicity index as defined in Peona et al. (2021):

$$\mathrm{Toxicity}\;\mathrm{index}=\;\frac{2n_{het}-\;2n_{hom}}{2n_{hom}}.$$

Here, 2*n*_het_ is equal to the number of diploid fl-LTRs in the heterogametic sex (i.e., 2 × autosomes + 1 × Z + 1 × W) and 2*n*_hom_ equals the number in the homogametic sex (2 × autosomes + 2 × Z). An index equal to 0 indicates no sex bias in toxicity, whereas a toxicity index >0 indicates female-biased toxicity and <0 indicates male-biased toxicity in the case of ZW snakes.

### Gene Expression and Characterization of W-Linked Genes

We quantified gene expression of W-linked genes, with the expectation that genes that have remained functional since recombination suppression are expressed in female tissues. We mapped mRNA-seq datasets for ovary and testes (NCBI SRA accession PRJNA477004) and for two male and female liver and kidney samples (10 samples total; supplementary table S2, Supplementary Material online) to the updated genome annotation using STAR v2.7.1a (Dobin et al. 2013) with the flags –outFilterMultimapNmax 1, –outFilterMismatchNmax 1, –outFilterMismatchNoverLmax 0.1, and –twoPassMode Basic, then quantified raw read counts using featureCounts v2.0.1 (Liao et al. 2013). Raw counts from featureCounts were used to assess whether genes on the W chromosome had “detectable” expression in female and male samples, defined as a raw count >0 in at least one sample of a given sex. Genes with detected expression in female samples and no detected expression in male samples were considered to have female-specific expression. To further assess female-specific expression, we performed pairwise comparisons between female and male samples using DEseq2 v1.30.1 (Love et al. 2014) and conducted *P*-value correction using IHW v1.18.0 (Ignatiadis et al. 2016) with baseMean expression as the covariate. Female-specific genes were defined as those with significant differential expression (IHW *P*-value <0.05) and higher expression in female samples compared with males (i.e., log_2_FoldChange >0). TPM-normalized counts were calculated in R and averaged across males and females to provide representative expression values for each sex. Heatmaps were generated using pheatmap v1.0.12 (Kolde 2012) in R.

We functionally annotated genes on the W chromosome using GO term analysis in WebGestaltR (Liao et al. 2019; https://github.com/bzhanglab/WebGestaltR), with W-linked genes as the foreground and annotated genes on other chromosomes as the background gene set. Using the overrepresentation analysis method, we selected the top 20 overrepresented terms from combined non-redundant biological process, cellular component, and molecular function GO-term databases, and a combined KEGG (Kanehisa and Goto 2000), Panther (Mi and Thomas 2009; Mi et al. 2021), Reactome (Jassal et al. 2020), and Wikipathway (Martens et al. 2021) pathway database. We tested for enrichment of W-linked genes for specific terms using Fisher’s exact tests and defined significantly overrepresented terms as those with *P*-values below 0.05 after FDR correction for multiple tests. We characterized protein classes for W-linked genes using PantherDB (Mi et al. 2019). This method was also used to characterize W-specific duplicated and translocated genes described below.

### Analysis of W-Linked Genes: Survival, Duplication, Selection, and Gene Conversion

Previous evidence suggests disproportionate survival of W-linked genes in the recent stratum (Schield et al. 2019). To test this hypothesis, we calculated a decay rate for stratum 1 (i.e., older strata) by subtracting the number of W genes from the number of Z genes present in stratum 1, divided by the inferred divergence time for the stratum. We compared this rate to one for the recent stratum to test whether genes have decayed at similar rates in the time since recombination suppression in both strata. To test for evidence of autosome-to-W chromosome translocations, we used a reciprocal best BLAST between W chromosome and *Anolis* CDS sequences. *Anolis* chromosome 6 is homologous to the colubroid ZW chromosomes, therefore we retained any hits to other chromosomes that also did not have a matched Z chromosome gametolog as candidate autosomal translocations.

We tested for W-specific gene duplications by mapping female resequencing data (*n* = 4; supplementary table S1, Supplementary Material online; Schield et al. 2021, 2022) to autosomes, the Z chromosome, and the W chromosome using BWA mem v0.7.17 (Li and Durbin 2009), after quality filtering reads using Trimmomatic v0.39 (Bolger et al. 2014) with settings described above. We calculated mean read depth per gene (within exon boundaries) using Mosdepth (Pedersen and Quinlan 2018) and determined the median among autosomal genes per female. We then quantified relative W-linked gene read depth by dividing mean depth per gene by the autosomal median in each female after accounting for haploidy of the W chromosome. This provided relative depths per gene scaled to a value of 1. We inferred copy-number as the mean of relative depth values observed across females per W-linked gene.

We compared rates of sequence divergence between prairie rattlesnake and five-pace viper Z and W chromosome orthologs, respectively, following the procedure described above for prairie rattlesnake ZW gametologs. Briefly, we identified sex-linked orthologs between the two species, then aligned orthologs using Clustal Omega (Sievers et al. 2011) and PAL2NAL (Suyama et al. 2006). We used the codon model in PAML (Yang 2007) to estimate *d*_N_, *d*_S_, and *d*_N_/*d*_S_ separately for the Z chromosome and W chromosome. We compared rates of nonsynonymous and synonymous evolution between the sex chromosomes to detect whether expected differences in the efficiency of natural selection are present on the female-specific W chromosome. Cases of gene conversion within genes on sex-limited chromosomes have been documented (e.g., Backström et al. 2005), along with an associated increase in GC bases due to GC-biased gene conversion. To test for evidence of gene conversion within W-linked genes (i.e., intrachromosomal recombination), we compared the proportion of GC bases at third codon positions (GC3) between matched Z and W gametologs in the prairie rattlesnake.

### Statistical Analysis

We performed statistical analyses comparing distributions of genomic variation and divergence in R (R Core Team 2017). Prior to performing pairwise comparisons between variables, we tested for normality of *d*_S_, *d*_N_/*d*_S_, GC content, CpG content, repeat content (including specific repeat element families), and GC3 distributions using Shapiro–Wilk tests. We further evaluated homogeneity of variances between distributions using Levene’s tests. All distributions rejected normality (*P*-values <0.05) with the exception of GC3. Accordingly, we tested for significant differences in pairwise comparisons of *d*_S_, *d*_N_/*d*_S_, GC content, CpG content, and repeat content distributions on the sex chromosomes and autosomes using Mann–Whitney *U* tests and tested for associations between variables using Spearman’s rank order correlation coefficients. We calculated partial correlation coefficients between GC content, CpG content, and repeat content while controlling for correlation among sets of variables using the “ppcor” R package (Kim 2015). Because GC3 distributions were not significantly different from a normal distribution, we performed pairwise comparisons using Welch’s two-sample *t*-tests. All distributions supported the hypothesis of equal variances (i.e., Levene’s test *P*-values >0.05) with the exception of *d*_S_ estimates between ZW gametologs among inferred evolutionary strata (see Results).

## Supplementary Material

evac116_Supplementary_Data

## Data Availability

The W chromosome assembly generated in this study is available from NCBI Genbank under accession PRJNA853338. RNAseq data generated in this study are available from the NCBI short-read archive under accession PRJNA477004. Gene and repeat annotations are available at https://github.com/drewschield/rattlesnake_w_chromosome/tree/main/resources/annotation. The computational workflow and analysis scripts are available at https://github.com/drewschield/rattlesnake_w_chromosome.
