# Correction to: The Rattlesnake W Chromosome: A GC-Rich Retroelement Refugium with Retained Gene Function Across Ancient Evolutionary Strata

**DOI:** 10.1093/gbe/evac154

**Published:** 2022-11-04

This is a correction to: Drew R Schield, Blair W Perry, Daren C Card, Giulia I M Pasquesi, Aundrea K Westfall, Stephen P Mackessy, Todd A Castoe, The Rattlesnake W Chromosome: A GC-Rich Retroelement Refugium with Retained Gene Function Across Ancient Evolutionary Strata, Genome Biology and Evolution, Volume 14, Issue 9, September 2022, evac116, https://doi.org/10.1093/gbe/evac116

The URL in the data availability sections has been replaced with:

https://github.com/drewschield/rattlesnake_w_chromosome/tree/main/resources/annotation

