# Supplementary material for: The Rattlesnake W Chromosome: A GC-Rich Retroelement Refugium with Retained Gene Function Across Ancient Evolutionary Strata
Source: Genome Biol Evol. 2022 Jul 22;14(9):evac116. doi: 10.1093/gbe/evac116 (PMC9447483; doi:10.1093/gbe/evac116)
Supplement: evac116_Supplementary_Data [file evac116_supplementary_data.zip › Supplementary Materials Captions.docx]

# Supplementary Materials Captions

**Supplementary Table S1**. Whole genome resequencing reads used in this study.

**Supplementary Table S2**. RNA-seq samples used for transcriptome assembly and differential expression analyses.

**Supplementary Table S3**. Divergence between Z and W gametologs. Gene names, Z chromosome start positions (bp), and inferred evolutionary strata are provided, along with GC3 measures for W and Z gametologs per pair, divergence (dS) between ZW gametologs in the prairie rattlesnake (*Crotalus*), and synonymous divergence and the ratio of nonsynonymous to synonymous divergence (dN/dS) between prairie rattlesnake and five-pace viper (*Deinagkistrodon*) W and Z gametologs, respectively.

**Supplementary Table S4**. GC content of autosomes and sex chromosomes for the prairie rattlesnake, birds, and mammals. Distributions of GC content on the W(Y) in each species were compared to the Z(X) and autosomes, respectively, using Mann-Whitney $\cup$ tests.

**Supplementary Table S5**. W chromosome repeat element composition annotated using RepeatMasker.

**Supplementary Table S6**. Expected and observed bp of repeat element classes on the autosomes, Z chromosome, and W chromosome used for refugium hypothesis tests.

**Supplementary Table S7**. TPM-normalized counts of gene expression for W-linked genes across tissues in females and males. Male gene expression was used to verify the presence of divergent gametologs between the sex chromosomes, or in cases of male gene expression, the presence of similar gametologs on the respective chromosomes. In both sexes, data from kidney and liver samples from two individuals were analyzed.

**Supplementary Table S8**. W-linked genes with evidence of translocation from ancestral autosomes. Each of the listed W gene annotations lacks an apparent Z gametolog and has homology with an *Anolis* gene on a scaffold other than Chromosome 6, the chromosome homologous to the ZW chromosomes in snakes.

**Supplementary Table S9**. Inferred copy number for W-linked genes based on female read mapping. Genes without a Z chromosome position did not have a matched Z gametolog.

**Supplementary Figure S1**. FastQC summary of 10x Genomics Chromium reads used in the female prairie rattlesnake genome assembly. **A**. Mean Phred quality scores per base. Green, yellow, and red shaded regions denote good, intermediate, and poor quality scores. **B**. Mean quality scores across sequencing reads. **C**. Per base sequence content. Data for the first 10-20 bp correspond to 10x Genomics barcodes used in sequencing and assembly steps. **D**. Proportion of GC bases across sequenced reads.

**Supplementary Figure S2.** Reanalysis of the female Indian cobra (*Naja naja*) scaffold previously reported as the W chromosome (Super-Scaffold_1000010). **A**. Male and female read depths in 100 kb sliding windows across Super-Scaffold_1000010, calculated as log_2_ of the read depth within each window divided by the median depth across autosomal scaffolds. The bottom panel shows the ratio of normalized male:female read depth, log_2_MF. Dashed lines in the top two panels are set at -2 to show roughly equal normalized coverage in both sexes. The dashed line in the bottom panel is set at 0, the expectation for autosomal regions. **B**. Dotplots showing homology between the Indian cobra Z chromosome and the rattlesnake (*Crotalus*) Z chromosome and *Anolis* chromosome 6 and between Super-Scaffold_1000010 and Indian cobra chromosome 6, rattlesnake chromosome 4, and *Anolis* chromosome 3. **C**. Normalized ratio of female:male read depth, log_2_FM, in 1 kb sliding windows on the three largest candidate Indian cobra W chromosome scaffolds identified in this study. Red dashed lines show log_2_FM = 1, the threshold used to identify candidate W-linked sequences. Grey regions depict gaps in assembled super-scaffolds.

**Supplementary Figure S3**. Distribution of annotated mdg4 retroelement lengths on autosomes (grey), the Z chromosome (green), and W chromosome (blue). ^***^ Denotes significant differences in mdg4 lengths based on Mann-Whitney $\cup$ tests.

**Supplementary Figure S4.** Gene expression of annotated W-linked genes, measured using log_10_ TPM normalized counts across female and male tissues. Mean values across female and male tissues are shown in the center. Gene names (or uncharacterized gene identifiers) are shown to the right, along with annotation of whether genes are within the recent or older strata or could not be assigned to 1:1 ZW gametolog positions.

**Supplementary Figure S5**. Pathways represented by W-linked genes. Bars depict numbers of genes falling into various pathway classifications. Bar color represents the specific pathway databases (KEGG, Reactome, and Wikipathway).

**Supplementary Figure S6.** GO terms, pathways, and protein classes represented by W-linked translocated genes (**A**-**B**) and genes with evidence of W-specific duplications (**C-D**). Bars depict numbers of genes falling into various GO term and pathway classifications. Bar color represents the specific GO term (Biological Process, Cellular Component, and Molecular Function) or pathway databases (KEGG, Reactome, and Wikipathway).
