# Supplementary material for: The Rattlesnake W Chromosome: A GC-Rich Retroelement Refugium with Retained Gene Function Across Ancient Evolutionary Strata
Source: Genome Biol Evol. 2022 Jul 22;14(9):evac116. doi: 10.1093/gbe/evac116 (PMC9447483; doi:10.1093/gbe/evac116)
Supplement: evac116_Supplementary_Data [file evac116_supplementary_data.zip › Supplementary_Appendix_06.23.22.docx]

# Supplementary Appendix

## Reanalysis of the reported Indian Cobra W chromosome

The Indian cobra (*Naja naja*) genome assembly included a reported 52.1 Mb W chromosome scaffold (Suryamohan et al. 2020). The scaffold was identified using mapping of male and female reads and a BLAST (Altschul et al. 1990) search of the W chromosome *CTNNB1* gametolog. Preliminary comparative analyses caused us to question whether this scaffold (‘Super-Scaffold_1000010’) is indeed W-linked, as patterns were instead consistent with expectations for autosomes. Further, previous methods used to identify the reported W chromosome scaffold may have produced misleading evidence of W-linkage for two reasons. First, it is unclear how male and female mappings were used to determine that the scaffold was the W chromosome and no comparison between the sexes was described. Lower coverage on the scaffold compared to others in males could produce spurious evidence of W-linkage without explicit comparison between the sexes, after accounting for the distribution of coverage within each sex, respectively. Second, *CTNNB1* homology on this scaffold is not necessarily evidence of W-linkage, as *CTNNB1* also maps to autosomal chromosome 6 in elapids (O’meally et al. 2010). We therefore performed reanalysis using multiple approaches to test whether the female Indian cobra Super-Scaffold_1000010 represents the W chromosome or an autosome.

First, we performed comparative mapping of male and female Indian cobra reads using bwa mem (Li and Durbin 2009), and calculated the ratio of male:female read depths (log_2_MF; see Methods for a detailed description of this calculation) across the scaffold in 100 kb sliding windows using Mosdepth (Pedersen and Quinlan 2018). Read depths for each sex were first normalized by the median depth across all scaffolds. Autosomal regions are expected to have log_2_MF = 0 because both sexes have the same ploidy for autosomes. Accordingly, we expected that a W-linked region would have significantly lower log_2_MF than the autosomal expectation, based on its presence in females and not males. In contrast, males and females have roughly equal normalized read depths and therefore equal copy-number across Super-Scaffold_1000010 (Supplementary Fig. S3A; mean log2MF = -0.046 $\pm$ 0.33; Mann-Whitney $\cup$ test between male and female read depth distributions, *p*-value = 0.49).

A second critical prediction for W-linkage on Super-Scaffold_1000010 is that it will have homology with the Indian cobra Z chromosome. Similarly, we would expect it to be homologous to the Z chromosome of other colubroid snakes (e.g., prairie rattlesnake) and chromosome 6 of *Anolis*. For comparative purposes, we first established homology between the Indian cobra Z chromosome and the prairie rattlesnake Z chromosome and *Anolis* chromosome 6 using MashMap (Jain et al. 2018), specifying the one-to-one option for each search. These analyses support the expected co-linear synteny between the Z chromosomes of the two snakes species and chromosome 6 in *Anolis* (Supplementary Fig. S3B). As such, we would expect Super-Scaffold_1000010 to be homologous to *Anolis* chromosome 6, the prairie rattlesnake Z chromosome, and most importantly the Indian cobra Z chromosome if it represents the W chromosome. Instead, Super-Scaffold_1000010 shows strong evidence of autosomal synteny, with co-linear homology to a region of Indian cobra chromosome 6, which is syntenic with prairie rattlesnake chromosome 4 and *Anolis* chromosome 3 (Supplementary Fig. S3B).

Similarly, we would expect annotated genes on Super-Scaffold_1000010 to have orthologous genes on the Z chromosome (i.e., ZW gametologs). To test this prediction, we performed a reciprocal best BLAST search between genes on Super-Scaffold_1000010 and the male Indian cobra reference. Consistent with broad evidence for homology between Super-Scaffold_1000010 and chromosome 6, 94% of genes on the scaffold had reciprocal best hits on chromosome 6; only 3 genes had best hits on the Z chromosome.

Together, these lines of evidence are consistent with Indian cobra Super-Scaffold_1000010 representing a region of autosomal chromosome 6 and being previously misidentified as the W chromosome.

To identify candidate W-linked scaffolds in the female Indian cobra assembly, we followed our methodology used to identify W chromosomes scaffolds in the rattlesnake (see Methods for details) using read depth information from female and male Indian cobras described above. Briefly, we defined candidate W-linked female scaffolds as those with mean log_2_FM > 1 and which did not have sequence similarity to autosomes in the male Indian cobra assembly. This procedure identified 6,242 candidate W-linked female scaffolds with a total length of 35.9 Mb (Supplementary Data).

## Supplementary References

Altschul, S. F., W. Gish, W. Miller, E. W. Myers, and D. J. Lipman. 1990. Basic local alignment search tool. *J Mol Biol* 215:403–410.

Jain, C., S. Koren, A. Dilthey, A. M. Phillippy, and S. Aluru. 2018. A fast adaptive algorithm for computing whole-genome homology maps. *Bioinformatics* 34:i748–i756.

Li, H., and R. Durbin. 2009. Fast and accurate short read alignment with Burrows-Wheeler transform. *Bioinformatics* 25:1754–1760.

O’meally, D., H. R. Patel, R. Stiglec, S. D. Sarre, A. Georges, J. A. M. Graves, and T. Ezaz. 2010. Non-homologous sex chromosomes of birds and snakes share repetitive sequences. *Chromosom Res* 18:787–800.

Pedersen, B. S., and A. R. Quinlan. 2018. Mosdepth: quick coverage calculation for genomes and exomes. *Bioinformatics* 34:867–868.

Suryamohan, K., S. P. Krishnankutty, J. Guillory, M. Jevit, M. S. Schröder, M. Wu, B. Kuriakose, O. K. Mathew, R. C. Perumal, and I. Koludarov, L. D. Goldstein, K. Senger, M. Davis Dixon, D. Velayutham, D. Vargas, S. Chaudhuri, M. Muraleedharan, R. Goel, Y. J. Chen, A. Ratan, P. Liu, B. Faherty, G. de la Rosa, H. Shibata, M. Baca, M. Sagolla, J. Ziai, G. A. Wright, D. Vucic, S. Mohan, A. Antony, J. Stinson, D. S. Kirkpatrick, R. N. Hannoush, S. Durinck, Z. Modrusan, E. W. Stawiski, K. Wiley, T. Raudsepp, R. Manjunatha Kini, A. Zachariah, and S. Seshagiri. 2020. The Indian cobra reference genome and transcriptome enables comprehensive identification of venom toxins. *Nat Genet* 52:106–117.
