# Supplementary material for: The Rattlesnake W Chromosome: A GC-Rich Retroelement Refugium with Retained Gene Function Across Ancient Evolutionary Strata
Source: Genome Biol Evol. 2022 Jul 22;14(9):evac116. doi: 10.1093/gbe/evac116 (PMC9447483; doi:10.1093/gbe/evac116)
Supplement: evac116_Supplementary_Data [file evac116_supplementary_data.zip › SuppTables_combined.pdf]

Supplementary Table S1. Whole genome resequencing reads used in this study.

Species	Sample ID	Sex	Read type	Number of reads	Experiment*	References
Thamnophis elegans	–	female	100 bp paired end	96,318,634	B	Vicoso et al. 2013
Thamnophis elegans	–	male	100 bp paired end	113,467,002	B	Vicoso et al. 2013
Deinagkistrodon acutus	–	female	150 bp paired end	400,914,792	B	Yin et al. 2016
Deinagkistrodon acutus	–	male	150 bp paired end	396,849,666	B	Yin et al. 2016
Sistrurus miliarius	–	female	100 bp paired end	105,176,638	B	Vicoso et al. 2013
Sistrurus miliarius	–	male	100 bp paired end	93,965,472	B	Vicoso et al. 2013
Crotalus viridis	CV0011	female	150 bp paired end	629,971,926	A, B, C	Schield et al. 2019
Crotalus viridis	CV0629	female	150 bp paired end	325,201,850	C	Schield et al. 2022
Crotalus viridis	CV0646	female	150 bp paired end	229,649,748	C	Schield et al. 2022
Crotalus viridis	CV0650	female	150 bp paired end	559,341,904	C	Schield et al. 2022
Crotalus viridis	CV0007	male	150 bp paired end	493,612,334	A, B	Schield et al. 2019

*Experiments: A = W chromosome scaffold identification, B = comparative mapping, C = gene duplications.

Supplementary Table S2. RNA-seq samples used for transcriptome assembly and differential expression analyses.

Tissue	Individual ID	Sex	Analysis	Accession
heart	CV10	Female	transcriptome	SRR19928820
heart	CV11	F	transcriptome	SRR19928819
heart	CV8	F	transcriptome	SRR19928818
heart	CV9	F	transcriptome	SRR19928817
kidney	CV10	F	transcriptome	SRR19928816
kidney	CV11	F	transcriptome	SRR19928815
kidney	CV3	F	transcriptome and differential expression	SRX4274153
kidney	CV8	F	transcriptome and differential expression	SRX4274152
kidney	CV9	F	transcriptome	SRR19928814
kidney	CV5	M	differential expression	SRX4274149
kidney	CV6	M	differential expression	SRX4274148
liver	CV10	F	transcriptome	SRR19928813
liver	CV11	F	transcriptome	SRR19928812
liver	CV3	F	transcriptome and differential expression	SRX4274155
liver	CV8	F	transcriptome and differential expression	SRX4274154
liver	CV9	F	transcriptome	SRR19928811
liver	CV5	M	differential expression	SRX4274151
liver	CV6	M	differential expression	SRX4274150
ovary	Cvv35	F	differential expression	SRX4274136
small intestine	CV11	F	transcriptome	SRS4900631
small intestine	CV8	F	transcriptome	SRS4900639
small intestine	CV9	F	transcriptome	SRS4900638
testes	Cvv29	M	differential expression	SRX4274142

Supplementary Table S3. Divergence between Z and W gametologs. Gene names, Z chromosome start positions (bp), and inferred evolutionary strata are provided, along with GC3 measures for W and Z gametologs per pair, divergence (d_S) between ZW gametologs in the prairie rattlesnake (*Crotalus*), and synonymous divergence and the ratio of nonsynonymous to synonymous divergence (d_N/d_S) between prairie rattlesnake and five-pace viper (*Deinagkistrodon*) W and Z gametologs, respectively.

					Crotalus		***Crotalus* versus *Deinagkistrodon***			
**Gene**	**Z position**	**Stratum**	**GC3 (W)**	**GC3 (Z)**	**ZW d_S**		**d_S (W)**	**d_N/d_S (W)**	**d_S (Z)**	**d_N/d_S (Z)**
GARS	2,476,541	1	0.554	0.571	0.623		0.5205	0.2922	0.133	0.3122
RBM33	6,652,235	1	0.471	0.391	0.2209		0.2154	0.5469	0.085	0.3557
LARP4B	9,429,493	1	0.365	0.372	0.452		0.2602	0.9132	0.095	0.5308
CUL2	13,072,015	1	0.469	0.444	0.0645		0.1105	0.001	–	–
EPC1	14,838,677	1	0.374	0.356	0.0318		0.0491	1.005	0.067	0.5733
ZEB1	15,120,182	1	0.286	0.293	0.1811		0.1561	0.4807	0.058	0.2485
WAC	16,279,701	1	0.231	0.216	0.9715		0.2977	0.3075	0.048	0.246
TMBIM7P	18,211,983	1	0.307	0.82	–		0.2379	0.1454	–	–
NMT2	20,364,506	1	0.422	0.524	–		0.2115	0.2088	0.207	0.4099
BMI1	24,004,966	1	0.448	0.534	0.5564		0.0333	0.1811	0.213	0.4574
PTF1A	24,384,785	1	0.643	0.688	0.3677		0.0676	0.4593	0.092	0.1173
UMAD1	27,718,821	1	0.366	0.503	–	–	–	–	–	–
SP4	33,937,051	1	0.258	0.264	0.195		0.0185	0.6534	0.033	0.3807
SP4	33,953,392	1	0.284	0.314	0.1837		–	–	–	–
HOXA1	36,544,550	1	0.673	0.556	–	–	–	–	0.052	0.101
HOXA2	36,550,838	1	0.585	0.573	0.1266		0.0435	0.6581	0.042	0.0371
HOXA6	36,597,885	1	0.784	0.667	0.5189		0.0296	0.2911	0.05	0.6959
HOXA9	36,617,150	1	0.751	0.724	0.1196		0.1599	0.7181	0.06	0.4401
DAZL	38,445,412	1	0.331	0.311	0.1967		0.2103	0.294	0.056	0.5386
NA	40,973,743	1	0.488	0.434	1.3465		–	–	–	–
MLV	40,974,268	1	0.419	0.487	0.4457		–	–	–	–
STT3B	45,446,651	1	0.346	0.346	0.3512		0.0405	0.1857	0.026	0.001
SNRK	46,642,535	1	0.256	0.274	0.3763		0.1136	0.522	0.058	0.0182
LOC106551782	48,075,357	1	0.367	0.398	0.273		–	–	–	–
TMEM108	48,228,093	1	0.416	0.404	–		–	–	0.104	0.7261
CCR5	48,423,830	1	0.385	0.601	–		0.1018	0.0969	0.055	1.193
CTNNB1	49,525,535	1	0.333	0.343	0.2642		0.0111	0.001	0.061	0.0748
PSMB3	74,103,894	1	0.434	0.451	0.4715		0.4884	0.3475	0.015	0.2795
PIP4K2B	74,113,048	1	0.364	0.424	0.5142		0.2307	0.5257	0.052	0.0192
CYP2C5	76,177,000	1	0.459	0.459	–		0.0848	0.9085	–	–
ANKRD17	76,427,468	1	0.319	0.714	–		0.1138	0.0854	–	–
GPATCH8	77,979,781	1	0.4	0.431	0.2196		0.0285	0.5868	0.0468	0.1702
FAM171A2	78,127,648	1	0.491	0.516	0.2443		0.2374	0.2242	0.0469	0.0609
PSMD11	79,118,366	1	0.469	0.381	0.3277		0.073	0.9653	0.0544	0.0388
TLK2	79,712,309	1	0.324	0.324	0.3312		0.3345	0.2214	0.0893	0.1411
UBTF	80,659,852	1	0.513	0.531	0.2379		0.0214	0.0623	0.0956	0.0344
FBXL20	81,511,122	1	0.528	0.547	0.267		0.2299	0.0718	0.1641	0.4342
CDK12	81,609,785	1	0.376	0.391	0.2381		0.0273	0.3508	0.0458	0.1957
LCA5	82,689,887	1	0.419	0.392	0.1181		0.7108	0.6549	0.4052	0.7913
MSL1	82,981,183	1	0.407	0.452	0.3266		0.0816	0.6087	0.0551	0.0835
RARA	83,517,645	1	0.488	0.504	0.1423		0.1706	0.2141	–	–
STAT5B	86,441,671	1	0.49	0.504	0.2416		–	–	0.0622	0.0268
KPNB1	87,892,282	1	0.402	0.352	0.6688		0.0125	0.001	0.0635	0.1299
SP2	88,396,988	1	0.44	0.442	0.2827		0.0716	0.6348	0.0793	0.1707
NFE2L1	88,670,286	1	0.453	0.481	0.2426		0.025	0.2599	0.0316	0.1601
CBX1	88,702,830	1	0.443	0.494	0.874		0.9188	0.3187	0.1134	0.001
HOXB3	89,380,338	1	0.675	0.701	0.2347		0.1064	0.6538	0.0725	0.075
HOXB6	89,440,816	1	0.709	0.619	0.7029		0.3121	0.7533	0.4623	0.4995
HOXB8	89,459,248	1	0.71	0.731	0.1132		0.0562	0.7151	0.4626	0.6367
UBE2Z	89,730,244	1	0.482	0.518	0.2756		–	–	0.0564	0.0667
ZNF652	90,035,554	1	0.314	0.392	0.3341		0.3573	0.1506	0.0399	0.0689

SPOP	90,532,934	1	0.458	0.479	0.1562	0.0527	0.001	0.0374	0.0329
KAT7	90,759,908	1	0.411	0.449	0.2494	0.0471	0.1853	0.0739	0.0281
HIST1H1A	91,479,689	1	0.571	0.859	–	–	–	0.0494	0.6156
COL1A1	91,497,833	1	0.526	0.49	0.4766	0.2365	0.5255	0.5778	0.4927
HDAC5	92,334,440	1	0.44	0.468	0.1521	0.0786	0.0528	0.0521	0.0705
SCAF1	92,837,626	1	0.435	0.464	0.2847	0.0455	1.0895	0.0992	0.4088
RRAS	92,879,514	1	0.554	0.585	0.4015	0.4892	0.6288	0.0882	0.0242
PRR12	92,897,746	1	0.564	0.575	0.2484	0.0328	0.2428	0.0787	0.1413
AP2A1	93,239,319	1	0.517	0.544	0.2331	0.0109	0.7663	0.0809	0.2127
SNRNP70	93,458,083	1	0.462	0.516	0.3028	0.2663	0.3734	0.0491	0.0414
PPFIA3	93,527,902	1	0.519	0.546	0.7686	0.4397	0.3838	0.0691	0.1459
TRIM28	95,035,682	1	0.456	0.465	0.237	0.2421	0.3668	0.0769	0.0888
ZSCAN16	98,343,344	2	0.471	0.668	–	0.2999	0.383	0.0354	0.1393
ZNF219	98,510,960	2	0.495	0.518	0.0682	0.174	0.8914	–	–
ZNF268	98,535,594	2	0.341	0.352	0.0446	0.0848	0.4397	0.1579	0.4642
MFAP4	99,720,703	2	0.478	0.478	0.0861	0.0186	0.1226	0.196	0.4381
LOC106548453	99,846,619	2	0.208	0.233	0.0878	0.081	0.2512	0.1731	0.2619
PRSS27	100,149,893	2	0.44	0.44	0.07	1.0029	0.5765	–	–
PAQR4	100,400,887	2	0.583	0.578	0.0597	0.0579	0.707	0.056	0.2387
APOBR	100,797,148	2	0.374	0.377	0.0736	0.0943	0.6427	0.0994	0.4092
ATP2A1	100,982,867	2	0.633	0.614	0.117	0.1124	0.0489	0.1284	0.2403
SH2B1	101,055,049	2	0.513	0.496	0.0438	0.0779	0.1541	0.0628	0.1489
TBC1D17	101,342,258	2	0.592	0.636	0.1039	0.0965	0.1521	0.102	0.088
GYS1	101,438,445	2	0.546	0.474	0.3168	0.0429	0.0658	0.0722	0.0274
FTL	101,478,377	2	0.679	0.655	0.0996	0.1528	0.1609	0.0699	0.1022
DNAAF3	102,286,382	2	0.65	0.664	0.0705	0.07	0.3021	0.1102	0.1581
PTPRH	102,356,675	2	0.543	0.543	0.0413	0.072	0.5248	0.157	0.6842
PPP6R1	102,450,425	2	0.462	0.434	0.3216	0.0473	0.7372	0.1478	0.4726
HSPBP1	102,507,380	2	0.627	0.5	0.3284	0.2828	0.9098	0.0659	0.2013
TMEM150B	102,645,603	2	0.606	0.543	0.1426	0.1549	0.2498	0.0599	0.4876
EPS8L1	102,988,692	2	0.556	0.511	0.1844	0.095	1.1222	0.2206	0.6689
KCNJ14	103,238,334	2	0.577	0.584	0.0413	0.026	0.6094	0.0792	0.1063
CYTH2	103,245,655	2	0.697	0.68	0.3305	0.2639	0.4143	0.0761	0.0591
TLR13	103,277,956	2	0.487	0.49	0.0657	0.0723	0.2039	0.0863	0.3432
LMTK3	103,359,411	2	0.504	0.504	–	0.0716	0.1451	0.0757	0.1498
CACNG7	103,482,560	2	0.593	0.617	0.1309	0.0428	0.001	0.0685	0.001
CACNG8	103,538,585	2	0.618	0.648	0.173	0.0976	0.0278	0.135	0.0103
CCDC114	103,736,700	2	0.532	0.519	–	0.0891	0.3822	0.1395	0.1744
PRPF31	104,144,386	2	0.523	0.51	0.1092	0.5955	0.7057	0.1532	0.343
TFPT	104,158,398	2	0.473	0.491	0.1451	0.1545	0.7616	0.0604	0.2494
OSCAR	104,174,068	2	0.607	0.571	0.093	0.1335	0.5106	0.0887	0.2996
SHISA7	104,290,908	2	0.573	0.58	0.0729	0.0803	0.1044	0.0759	0.164
UBE2S	104,335,372	2	0.612	0.6	0.168	0.1895	0.6466	0.0972	0.0435
RCVRN	104,360,810	2	0.562	0.575	0.2336	0.258	0.7372	0.1272	0.0848
RASIP1	104,548,230	2	0.516	0.645	–	–	–	0.1564	0.5448
IZUMO1	104,686,082	2	0.509	0.527	0.2095	–	–	–	–
SULT2B1	104,763,789	2	0.408	0.422	0.4585	0.2712	1.0397	0.0811	0.1957
ISOC2	104,936,777	2	0.563	0.549	0.1085	0.1051	1.0268	0.0272	0.2436
MYH11	104,952,866	2	0.621	0.629	0.1201	0.0841	0.0959	0.0792	0.0798
EPN1	105,074,770	2	0.526	0.516	0.3171	0.2038	0.6947	0.2642	0.5759
U2AF2	105,112,978	2	0.522	0.522	0.2932	0.2681	0.8667	0.0891	0.3027
ZNF726	105,206,563	2	0.538	0.538	0.0375	0.0683	0.3099	0.0848	0.1832
SACS	105,235,849	2	0.503	0.517	0.1034	0.0978	0.4324	0.0911	0.2997
H4C1	105,318,166	2	0.603	0.513	0.1409	0.1037	0.231	0.0493	0.001
TBRG1	105,389,554	2	0.577	0.582	0.0643	0.1055	0.7029	0.091	0.2459
CORO1A	105,450,385	2	0.573	0.584	0.1347	0.0924	0.0552	0.1116	0.0487
GDPD3	105,512,448	2	0.52	0.469	0.3818	0.3598	0.8624	0.0709	0.3752
YPEL3	105,531,125	2	0.461	0.487	0.2506	0.2788	0.3836	0.0848	0.001
ALDOA	105,582,679	2	0.628	0.612	0.1096	0.2282	0.153	0.1111	0.0662

MVP	105,631,040	2	0.453	0.435	0.2887	0.3224	0.456	0.0866	0.1501
MAZ	105,680,423	2	0.461	0.47	0.0632	0.0401	0.3369	0.1344	0.6792
PRRT2	105,690,065	2	0.601	0.601	0.0897	0.0837	0.217	0.1233	0.4269
CDIPT	105,710,861	2	0.565	0.548	0.0275	0.0417	0.5526	0.1107	0.6259
KIF22	105,835,326	2	0.519	0.496	0.1813	0.0387	0.7829	0.0991	0.377
PAGR1	105,856,879	2	0.493	0.483	0.0786	0.0547	0.4662	0.0869	0.0905
ASPHD1	105,865,513	2	0.566	0.548	0.05	0.0504	0.1393	0.0328	0.0857
SEZ6L2	105,873,042	2	0.562	0.598	0.0932	0.0798	0.0748	0.0862	0.0708
INO80E	105,967,889	2	0.57	0.463	0.6348	0.3754	0.8815	0.1605	0.2836
GJB2	106,198,960	2	0.502	0.533	0.0899	0.0487	0.6106	0.0692	0.347
THOC6	106,257,774	2	0.535	0.479	0.1196	0.1723	0.2896	0.1081	0.224
HCFC1R1	106,267,800	2	0.525	0.505	0.0304	0.0768	0.2323	0.0731	0.2488
RAB35	106,273,569	2	0.717	0.717	–	1.4025	0.6431	0.3227	0.6034
TBC1D10B	106,457,061	2	0.604	0.588	0.2508	0.1754	0.6662	0.1464	0.2175
CA4	106,793,422	2	0.513	0.763	–	–	–	–	–

Supplementary Table S4. GC content of autosomes and sex chromosomes for the prairie rattlesnake, birds, and mammals. Distributions of GC content on the W(Y) in each species were compared to the Z(X) and autosomes, respectively, using Mann-Whitney ∪ tests.

**Species**	**Mean auto**[a]	**SD auto**	**Mean Z(X)**	**SD Z(X)**	**Mean W(Y)**	**SD W(Y)**
Prairie Rattlesnake (*Crotalus viridis*)	0.364	0.041	0.368	0.041	0.439^{***}	0.041
Zebra Finch (*Taeniopygia guttata*)	0.343	0.064	0.306	0.090	0.271^{***}	0.084
Chicken (*Gallus gallus*)	0.342	0.080	0.302	0.086	0.227^{***}	0.080
Human (*Homo sapiens*)	0.254	0.106	0.229	0.091	0.106^{***}	0.130
House Mouse (*Mus musculus*)	0.278	0.100	0.204	0.098	0.015^{***}	0.047

[a]auto = autosomal. $^{***}p < 2.2 \times 10^{-16}$.

Supplementary Table S5. W chromosome repeat element composition annotated using RepeatMasker.

**Repeat classification**	**Number of elements**	**Length masked (bp)**	**Percent of W chromosome**
**Total masked**	60535	19,173,370	81.15
**Total interspersed repeats**	47370	18,502,470	76.35
**Retroelements**	34042	15,363,834	67.93
SINEs	1621	203,452	0.86
Alu	0	0	0.00
MIRs	504	70,182	0.30
Other SINEs	1117	133,270	0.56
LINEs	15582	5,685,119	24.06
CR1-Like	6927	1,955,695	8.28
CR1/L3	5056	1,324,723	5.61
L2	626	209,522	0.89
Rex	285	99,384	0.42
R1/LOA/Jockey	54	5,642	0.02
R2/R4/NeSL	395	118,143	0.50
RTE/Bov-B	2745	805,524	3.41
L1/CIN4	4275	2,483,273	10.51
Other LINEs	2156	638,908	1.34
Other nonLTR	615	122,807	0.52
DIRS	1243	877,401	3.71
PLEs	2178	501,763	2.12
LTR elements	12803	7,973,292	33.75
BEL/Pao	714	615	0.00
Ty1/Copia	984	12,803	0.05
mdg4	7383	5,554,069	23.51
Retroviral	2413	1,098,859	4.65
ERVL	1	14	0.00
ERVL-MaLRs	0	0	0.00
ERV_classI	1508	718,408	3.04
ERV_classII	904	380,437	1.61
Other LTR	1309	1,306,946	5.53
**DNA transposons**	13328	2,370,542	10.03
hobo-Activator	6626	1,168,386	4.95
Tc1-IS630-Pogo	3619	764,170	3.23
En-Spm	291	41,752	0.18
MuDR-IS905	152	26,278	0.11
PiggyBac	44	2,464	0.01
Tourist/Harbinger	523	53,390	0.23
P elements	41	6,070	0.03
Rolling-circles	106	14,581	0.06
SPIN	172	17,082	0.07
Other DNA	1754	276,369	1.17
**Unclassified**	4222	768,094	3.25
**Total interspersed repeats**		18,039,222	76.35
Small RNA	71	6,023	0.03
Satellites	66	10,893	0.05
Simple repeats	7321	545,542	2.31
Low complexity	1485	108,442	0.46

Supplementary Table S6. Expected and observed bp of repeat element classes on the autosomes, Z chromosome, and W chromosome used for refugium hypothesis tests.

Element	Total Repeats	ERVs	mdg4	L1 LINEs	CR1 LINEs	fl-LTRs
Total bp	512,000,930	1,596,762	14,580,722	13,471,915	41,133,081	102,295,223
Expected autosome bp	457,774,624.31	1,427,648.04	13,036,469.55	12,045,096.93	36,776,653.32	91,461,078.55
Observed autosome bp	437,748,675	825,697	9,846,145	10,015,653	36,542,683	87,719,901
Expected Z chromsome bp	44,501,057.95	138,784.12	1,267,297.61	1,170,924.57	3,575,121.67	8,891,088.63
Observed Z chromosome bp	54,041,723	235,896	2,379,345	1,997,066	3,786,887	11,460,212
Expected W chromosome bp	9,725,247.73	30,329.84	276,954.84	255,893.50	781,306.01	1,943,055.82
Observed W chromosome bp	20,210,532	535,169	2,355,232	1,459,196	803,511	3,116,708
df	2	2	2	2	2	2
χ^2	14,226,220.66	8,724,788.90	17,352,014.47	6,583,173.63	14,663.08	1,604,306.02

ERVs = endogenous retroviruses; fl-LTRs = full-length LTR retrotransposons.

Supplementary Table S7. TPM-normalized counts of gene expression for W-linked genes across tissues in females and males. Male gene expression was used to verify the presence of divergent gametologs between the sex chromosomes, or in cases of male gene expression, the presence of similar gametologs on the respective chromosomes. In both sexes, data from kidney and liver samples from two individuals were analyzed.

	Female						Male					
Gene	ovary	kidney 1	kidney 2	liver 1	liver 2	Mean	testes	kidney 1	kidney 2	liver 1	liver 2	Mean
ZSCAN16	67.89	12.56	14.30	6.95	4.30	21.20	118.39	9.43	19.76	11.05	5.93	32.91
ZNF219	2.53	0.00	0.49	0.00	0.53	0.71	0.00	0.00	0.00	0.00	0.00	0.00
ZNF268	0.00	0.00	0.00	0.00	0.00	0.00	0.00	0.00	0.00	0.00	0.00	0.00
MFAP4	1.78	6.34	2.48	0.00	0.89	2.30	0.31	0.00	0.41	0.00	0.00	0.14
ERV3-1	0.00	0.00	0.00	0.00	0.00	0.00	1.41	0.00	0.00	0.00	0.00	0.28
PRSS27	0.00	0.00	0.00	0.00	0.00	0.00	0.00	0.00	0.00	0.00	0.00	0.00
PAQR4	1.22	0.00	0.00	0.00	0.00	0.24	0.00	0.00	0.00	0.00	0.00	0.00
APOBR	0.00	0.41	0.57	1.25	0.00	0.45	0.00	0.00	0.00	0.00	0.00	0.00
ATP2A1	1.73	0.00	0.00	0.00	0.00	0.35	0.00	0.00	0.00	0.00	0.00	0.00
SH2B1	32.61	3.96	3.39	2.24	2.91	9.02	0.00	0.00	0.00	0.00	0.00	0.00
TBC1D17	2.63	0.00	0.00	0.00	0.00	0.53	1.13	0.00	1.51	1.06	0.00	0.74
GYS1	14.25	3.03	3.11	0.00	0.00	4.08	0.00	0.00	0.00	0.00	0.00	0.00
FTL	6.70	3.80	228.61	24.38	51.59	63.02	11.52	77.39	84.62	41.23	53.84	53.72
DNAAF3	3.81	6.49	6.65	2.45	1.19	4.12	0.00	0.00	0.00	0.00	0.00	0.00
PTPRH	0.00	0.00	0.00	0.52	0.00	0.10	0.00	0.00	0.00	0.00	0.00	0.00
PPP6R1	27.78	25.11	18.17	17.83	10.83	19.94	0.00	0.00	0.00	0.00	0.00	0.00
HSPBP1	0.00	0.00	0.00	0.00	0.00	0.00	0.00	0.00	0.00	0.00	0.00	0.00
TMEM150B	2.18	0.00	0.00	0.00	0.00	0.44	0.00	0.00	0.00	0.00	0.00	0.00
EPS8L1	0.00	0.00	0.00	3.85	0.00	0.77	0.00	0.00	0.00	0.00	0.00	0.00
KCNJ14	0.00	0.00	0.21	0.00	0.00	0.04	0.00	0.00	0.00	0.00	0.00	0.00
CYTH2	8.86	0.00	0.00	0.00	0.00	1.77	0.00	0.00	0.00	0.00	0.00	0.00
TLR13	0.00	16.68	2.96	0.93	0.45	4.21	0.00	0.00	0.00	0.00	0.00	0.00
LMTK3	0.00	0.00	0.00	0.00	0.00	0.00	0.00	0.00	0.00	0.00	0.00	0.00
LMTK3	0.00	0.00	0.00	0.00	0.00	0.00	1.71	0.00	0.00	0.00	0.00	0.34
CACNG7	0.00	0.00	0.00	10.67	2.07	2.55	0.00	0.00	0.00	0.00	0.00	0.00
CACNG8	0.00	1.40	0.00	0.00	0.00	0.28	0.00	0.00	0.00	0.00	0.00	0.00
CCDC114	0.00	0.00	0.00	0.00	0.00	0.00	0.00	0.00	0.00	0.00	0.00	0.00
PRPF31	1.75	8.92	3.05	5.61	6.54	5.17	0.00	0.00	0.00	0.00	0.00	0.00
TFPT	8.74	7.44	9.70	7.65	5.45	7.80	0.00	0.00	0.00	0.00	0.00	0.00
OSCAR	0.00	0.00	0.00	0.00	0.00	0.00	0.00	0.00	0.00	0.00	0.00	0.00
SHISA7	0.00	0.00	0.00	0.00	0.00	0.00	0.00	0.00	0.00	0.00	0.00	0.00
UBE2S	126.80	9.92	8.47	3.74	5.46	30.88	0.00	0.00	0.84	0.00	0.00	0.17
RCVRN	1.65	0.00	0.00	0.00	0.00	0.33	0.00	0.00	0.00	0.00	0.00	0.00
RASIP1	0.00	0.00	0.00	1.50	0.00	0.30	0.00	0.00	0.00	0.00	0.00	0.00
IZUMO1	0.00	0.00	0.00	0.00	0.00	0.00	0.00	0.00	0.00	0.00	0.00	0.00
SULT2B1	0.00	0.00	0.00	3.55	0.00	0.71	0.00	0.00	0.00	0.00	0.00	0.00
ISOC2	0.00	0.00	0.77	0.00	0.00	0.15	0.00	0.00	0.00	0.00	0.00	0.00
MYH11	2.94	0.36	1.22	0.00	0.00	0.90	1.45	0.00	0.00	0.00	0.00	0.29
EPN1	11.36	12.08	9.90	9.11	3.54	9.20	0.00	0.00	0.00	0.00	0.00	0.00
U2AF2	81.07	44.04	52.15	19.93	10.77	41.59	0.00	0.00	0.00	0.00	0.00	0.00
ZNF726	0.00	0.00	0.67	0.00	0.00	0.13	0.00	0.00	0.00	0.00	0.00	0.00
SACS	0.00	0.00	0.00	0.00	0.00	0.00	0.00	0.00	0.00	0.00	0.00	0.00
H4C1	0.00	0.00	0.00	0.00	0.00	0.00	0.00	0.00	0.00	0.00	0.00	0.00
TBRG1	0.00	0.00	0.00	0.00	0.00	0.00	0.00	0.00	0.00	0.00	0.00	0.00
CORO1A	23.21	0.00	1.07	0.24	1.15	5.13	0.00	0.00	0.00	0.59	0.00	0.12
GDPD3	0.00	0.00	0.00	0.00	0.00	0.00	0.00	0.00	0.00	0.00	0.00	0.00
YPEL3	13.26	0.84	0.00	1.26	0.00	3.07	0.00	0.00	0.00	0.00	0.00	0.00
ALDOA	0.00	1.78	0.00	0.00	0.00	0.36	1.80	0.00	0.00	0.00	0.00	0.36
MVP	0.00	0.00	0.00	0.00	0.00	0.00	0.00	0.00	0.00	0.00	0.00	0.00
MAZ	85.81	10.09	10.55	2.24	5.22	22.78	0.00	0.00	0.00	0.00	0.00	0.00
PRRT2	0.00	0.00	0.00	0.00	0.00	0.00	0.00	0.00	0.00	0.00	0.00	0.00
CDIPT	0.00	0.00	0.00	0.00	0.00	0.00	0.00	0.00	0.00	0.00	0.00	0.00
KIF22	1.61	0.00	0.00	0.00	0.00	0.32	0.00	0.00	0.00	0.00	0.00	0.00
PAGR1	55.57	7.88	8.46	3.40	4.13	15.89	0.00	0.00	0.00	0.00	0.00	0.00
ASPHD1	0.73	0.00	0.85	0.23	0.23	0.41	0.00	0.00	0.00	0.00	0.00	0.00
SEZ6L2	2.44	0.00	0.00	0.00	0.00	0.49	0.00	0.00	0.00	0.00	0.00	0.00
INO80E	26.63	0.00	1.82	4.02	10.75	8.64	0.00	0.00	0.00	0.00	0.00	0.00
GJB2	0.00	0.00	0.31	16.04	6.97	4.66	0.00	0.00	0.00	0.00	0.00	0.00
THOC6	1.38	0.00	0.80	0.00	0.00	0.44	0.00	0.00	0.00	0.00	0.00	0.00
HCFC1R1	15.81	7.34	2.92	5.53	2.24	6.77	0.00	1.59	0.00	5.19	0.43	1.44
RAB35	28.67	11.81	11.83	5.94	5.20	12.69	0.00	0.00	0.00	0.00	0.00	0.00
TBC1D10B	1.08	1.84	1.26	0.00	1.35	1.11	0.00	0.00	0.00	0.00	0.00	0.00
CA4	0.00	0.00	0.00	0.00	0.00	0.00	0.00	0.00	0.00	0.00	0.00	0.00
GARS	0.00	0.00	0.00	0.00	0.00	0.00	0.00	0.00	0.00	0.00	0.00	0.00
RBM33	14.93	0.00	1.09	0.00	1.17	3.44	0.00	0.00	0.00	0.00	0.00	0.00
LARP4B	2.81	2.40	2.45	1.36	0.00	1.80	0.00	0.00	0.00	0.00	0.00	0.00
CUL2	10.05	2.28	1.95	0.43	2.51	3.44	0.00	0.00	0.00	0.00	0.00	0.00
EPC1	13.00	4.98	3.02	2.09	2.84	5.19	0.00	0.00	0.00	0.00	0.00	0.00
ZEB1	0.00	0.41	1.39	0.62	0.30	0.54	0.00	0.00	0.00	0.00	0.00	0.00
WAC	100.80	4.77	13.02	1.20	3.50	24.66	0.00	0.00	0.00	0.00	0.00	0.00
TMBIM7P	0.00	0.00	0.00	0.00	0.00	0.00	0.00	0.00	0.00	0.00	0.00	0.00
NMT2	0.00	0.00	0.00	0.00	0.00	0.00	0.00	0.00	0.00	0.00	0.00	0.00
BMI1	38.20	26.74	28.12	8.90	11.54	22.70	0.00	0.00	0.18	0.00	0.00	0.04
PTF1A	1.02	0.87	0.00	0.00	0.00	0.38	0.00	0.00	0.00	0.00	0.00	0.00
UMAD1	0.00	0.00	0.00	0.00	0.00	0.00	0.00	0.00	0.00	0.00	0.00	0.00
SP4	2.15	0.46	0.31	0.00	0.00	0.58	0.00	0.00	0.00	0.00	0.00	0.00
SP4	5.21	0.00	0.38	0.00	0.41	1.20	0.00	0.00	0.00	0.00	0.00	0.00
HOXA1	0.00	2.76	0.94	0.00	0.00	0.74	0.00	0.00	0.00	0.00	0.00	0.00
HOXA2	0.00	3.91	1.34	0.00	0.00	1.05	0.00	0.00	0.00	0.00	0.00	0.00
HOXA6	0.00	0.00	0.00	0.00	0.00	0.00	0.00	0.00	0.00	0.00	0.00	0.00
HOXA9	0.00	0.00	0.00	0.00	0.00	0.00	0.00	0.00	0.00	0.00	0.00	0.00
DAZL	64.02	0.00	0.00	0.00	0.00	12.80	0.00	0.00	0.00	0.00	0.00	0.00
NA	0.00	0.00	0.00	0.00	0.00	0.00	0.00	0.00	0.00	0.00	0.00	0.00
MLV	0.00	0.00	0.00	0.00	0.00	0.00	0.00	0.00	0.00	0.00	0.00	0.00
STT3B	15.14	2.15	8.80	6.48	1.57	6.83	0.00	0.00	0.00	0.00	0.00	0.00
SNRK	2.48	0.53	0.72	2.39	1.94	1.61	0.00	0.00	0.00	0.00	0.00	0.00
LOC106551782	2.54	0.00	0.74	0.00	0.79	0.81	1.09	0.00	0.00	0.00	0.00	0.22
TMEM108	0.00	0.00	0.00	0.00	0.00	0.00	0.00	0.00	0.00	0.00	0.00	0.00
CCR5	0.00	0.00	0.00	0.00	0.00	0.00	0.00	0.00	0.00	0.00	0.00	0.00
CTNNB1	51.89	53.13	46.67	17.96	17.96	37.52	0.00	0.00	0.00	0.00	0.00	0.00
PSMB3	0.00	0.00	0.00	0.00	0.00	0.00	0.00	0.00	0.00	0.00	0.00	0.00

PIP4K2B	18.63	10.57	7.82	4.65	2.58	8.85	0.00	0.00	0.00	0.00	0.00	0.00
CYP2C5	0.00	1.60	3.28	0.00	10.56	3.09	19.39	0.00	2.16	0.00	0.00	4.31
ANKRD17	0.00	0.00	0.00	0.00	0.00	0.00	166.10	0.00	0.00	0.00	0.31	33.28
GPATCH8	0.00	0.37	0.13	0.00	0.00	0.10	0.00	0.00	0.00	0.00	0.00	0.00
FAM171A2	0.00	0.00	0.00	0.00	0.00	0.00	0.00	0.00	0.00	0.00	0.00	0.00
PSMD11	47.82	16.28	30.58	26.09	7.46	25.64	0.00	0.00	0.00	0.00	0.00	0.00
TLK2	0.00	6.41	4.38	3.87	0.00	2.93	0.00	0.00	0.00	0.00	0.00	0.00
UBTF	3.42	1.45	2.98	0.00	1.60	1.89	0.00	0.00	0.00	0.00	0.00	0.00
FBXL20	0.00	0.00	0.00	0.00	0.00	0.00	0.00	0.00	0.00	0.00	0.00	0.00
CDK12	77.39	7.87	7.34	6.48	3.15	20.45	0.00	0.00	0.00	0.00	0.00	0.00
LCA5	0.00	0.00	0.00	0.00	0.00	0.00	2.22	0.00	0.00	0.00	0.00	0.44
MSL1	78.65	15.57	7.44	3.52	5.71	22.18	0.00	0.00	0.00	0.00	0.00	0.00
RARA	14.34	0.00	0.00	0.00	0.00	2.87	0.00	0.00	0.00	0.00	0.00	0.00
STAT5B	1.87	0.00	0.00	0.00	0.00	0.37	0.00	0.00	0.00	0.00	0.00	0.00
KPNB1	86.42	9.43	9.02	1.42	4.15	22.09	0.00	0.00	0.00	0.00	0.00	0.00
SP2	0.68	0.87	1.58	0.66	0.21	0.80	0.00	0.00	0.00	0.00	0.00	0.00
NFE2L1	2.08	5.30	6.84	0.89	0.86	3.19	0.00	0.00	0.00	0.00	0.00	0.00
CBX1	269.14	23.19	17.83	21.87	14.88	69.38	0.00	0.00	0.00	0.00	2.05	0.41
HOXB3	0.00	18.23	15.45	0.00	0.00	6.74	0.00	0.00	0.00	0.00	0.00	0.00
HOXB6	0.00	1.68	0.00	0.00	0.00	0.34	0.00	0.00	0.00	0.00	0.00	0.00
HOXB8	50.40	6.35	6.51	0.00	0.00	12.65	0.00	0.00	0.00	0.00	0.00	0.00
UBE2Z	6.50	6.69	10.08	4.87	2.88	6.20	0.00	0.00	0.00	0.00	0.00	0.00
ZNF652	10.62	1.51	4.12	5.68	1.10	4.61	0.00	0.00	0.00	0.00	0.00	0.00
SPOP	5.31	0.00	2.06	0.00	3.31	2.14	0.00	0.00	0.00	0.00	0.00	0.00
KAT7	37.48	1.16	1.98	0.44	0.43	8.30	0.00	0.00	0.00	0.00	0.00	0.00
HIST1H1A	0.00	0.00	0.00	0.00	0.00	0.00	0.00	0.00	0.00	0.00	0.00	0.00
COL1A1	5.30	3.26	5.14	0.00	0.92	2.92	0.00	0.00	0.00	0.00	0.00	0.00
HDAC5	0.00	2.13	2.91	0.00	0.00	1.01	0.00	0.00	0.00	0.00	0.00	0.00
SCAF1	0.00	0.00	0.00	0.00	0.00	0.00	0.00	0.00	0.00	0.00	0.00	0.00
RRAS	0.00	0.00	0.00	0.00	0.00	0.00	0.00	0.00	0.00	0.00	0.00	0.00
PRR12	3.39	0.00	1.74	0.26	0.25	1.13	0.00	0.00	0.00	0.00	0.00	0.00
AP2A1	1.40	18.05	16.07	9.14	6.62	10.26	0.00	0.41	0.00	0.00	0.00	0.08
SNRNP70	159.19	121.10	110.09	80.51	93.33	112.84	0.00	0.00	0.00	0.00	0.00	0.00
PPFIA3	0.00	0.00	0.00	0.00	0.00	0.00	0.00	0.00	0.00	0.00	0.00	0.00
TRIM28	14.47	5.13	7.71	0.77	1.13	5.84	0.00	0.00	0.00	0.00	0.00	0.00
LOC106538011	1.59	0.00	0.00	0.00	0.00	0.32	0.00	0.00	0.00	0.00	0.00	0.00
KANSL1	1.88	0.00	0.36	0.00	0.39	0.53	0.00	0.00	0.00	0.00	0.00	0.00
NA	1.08	0.00	0.00	0.00	0.00	0.22	0.00	0.00	0.00	0.00	0.00	0.00
PRR12	0.00	0.00	2.23	0.00	0.00	0.45	0.00	0.00	0.00	0.00	0.00	0.00
LOC106551782	0.00	0.00	0.00	0.00	0.00	0.00	0.00	0.00	0.87	0.00	0.00	0.17
LOC106551782	1.27	0.00	0.00	0.00	0.00	0.25	1.09	0.00	0.00	0.00	0.00	0.22
NA	0.00	0.00	2.53	3.72	3.62	1.97	1.25	0.00	0.00	0.00	0.00	0.25
PPP6R1	20.70	2.94	4.01	4.43	0.00	6.42	0.00	0.00	0.00	0.00	0.00	0.00
LOC106547512	0.00	0.00	0.00	0.00	0.00	0.00	0.00	0.00	0.00	0.00	0.00	0.00
MLV	17.85	23.47	38.67	16.66	7.09	20.75	18.13	7.17	7.45	1.30	6.84	8.18
LOC106548453	0.00	0.00	0.00	0.00	0.00	0.00	0.00	0.00	0.00	0.00	0.00	0.00
ERV3-1	0.00	0.00	0.00	0.00	0.00	0.00	0.00	0.00	0.00	0.00	0.00	0.00
HA1F	0.00	14.88	10.17	1.40	1.36	5.56	15.03	9.67	21.33	5.26	7.90	11.84
SOX8	0.00	0.00	0.00	0.00	0.00	0.00	0.00	0.00	0.00	0.00	0.00	0.00
NA	0.00	0.00	0.00	0.00	0.00	0.00	0.00	0.00	0.00	0.00	0.00	0.00
NA	0.00	0.00	0.00	0.00	0.00	0.00	0.00	0.00	0.00	0.00	0.00	0.00
NA	0.00	0.00	0.00	0.00	0.00	0.00	0.00	0.00	0.00	0.00	0.00	0.00
LOC106548453	0.00	0.00	0.00	0.00	0.00	0.00	0.00	0.00	0.00	0.00	0.00	0.00
NA	0.00	0.00	0.00	0.00	0.00	0.00	0.00	0.00	0.00	0.00	0.00	0.00
NA	0.00	0.00	0.00	0.00	0.00	0.00	0.00	0.00	0.00	0.00	0.00	0.00
ZNF319	0.00	0.00	0.00	0.00	0.00	0.00	0.00	0.00	0.00	0.00	0.00	0.00
EPC1	16.14	16.78	27.11	9.21	13.43	16.53	0.00	0.00	0.00	0.00	0.00	0.00
NA	0.00	15.26	6.26	4.60	2.24	5.67	0.00	0.00	0.00	0.00	0.00	0.00
GYS1	20.19	0.00	23.48	0.00	6.30	9.99	0.00	0.00	0.00	0.00	0.00	0.00
NA	0.00	0.00	0.00	0.00	0.00	0.00	0.00	0.00	0.00	0.00	0.00	0.00
NA	0.00	0.00	0.00	0.00	0.00	0.00	0.94	0.00	0.00	0.00	0.00	0.19
RBM14	0.00	0.00	0.00	0.00	0.00	0.00	0.00	0.00	0.00	0.00	0.00	0.00
RBM14	0.00	0.00	0.00	0.00	0.00	0.00	0.00	0.00	0.00	1.94	0.00	0.39
FAM171A2	0.00	0.00	0.00	0.00	0.00	0.00	0.00	0.00	0.00	0.00	0.00	0.00
NA	0.00	0.00	0.36	0.00	0.00	0.07	0.00	0.00	0.00	0.00	0.38	0.08
NA	0.00	0.00	0.00	0.00	0.00	0.00	0.00	0.00	0.00	0.00	0.00	0.00
NA	0.00	0.00	0.00	0.00	0.00	0.00	1.96	0.00	0.00	0.00	0.00	0.39
LOC106547512	0.00	0.00	0.00	0.00	0.00	0.00	0.00	0.00	0.00	0.00	0.00	0.00
LOC106548453	0.00	0.00	0.00	0.00	0.00	0.00	0.00	0.00	0.00	0.00	0.00	0.00
NA	0.00	0.00	0.00	0.00	0.00	0.00	0.00	0.00	0.00	0.00	0.00	0.00
TRIM28	29.87	15.89	13.02	7.19	5.83	14.36	0.00	0.00	0.00	0.00	0.00	0.00
H2-Q9	2.15	1.83	0.00	1.38	0.00	1.07	9.22	30.06	1.23	12.05	3.88	11.29
NA	0.00	0.00	0.00	0.00	0.00	0.00	0.00	0.00	0.00	0.00	0.00	0.00

Supplementary Table S8. W-linked genes with evidence of translocation from ancestral autosomes. Each of the listed W gene annotations lacks an apparent Z gametolog and has homology with an *Anolis* gene on a scaffold other than Chromosome 6, the chromosome homologous to the ZW chromosomes in snakes.

Gene	W chromosome annotation	*Anolis* chromosome homology
SLC22A7	maker-scaffold-W404072-augustus-gene-0.1	Chromosome 2
OR52D1	augustus_masked-scaffold-W393371-processed-gene-0.0	Chromosome Un0828
VWA5A	maker-scaffold-Wagouti77-augustus-gene-0.1	Chromosome Un0061
LOC103280460	augustus_masked-scaffold-W285958-processed-gene-0.0	Chromosome Un0208
MDFI	augustus_masked-scaffold-W391774-processed-gene-0.0	Chromosome 4
ZNF319	augustus_masked-scaffold-W283894-processed-gene-0.0	Chromosome Un0347
LOC103281823	maker-scaffold-W361845-augustus-gene-0.1	Chromosome Un0983
H2Q9	augustus_masked-scaffold-W298664-processed-gene-0.0	Chromosome Un1929
LOC100557651	maker-scaffold-W382211-augustus-gene-0.1	Chromosome Un0641
TMEM143	maker-scaffold-Wagouti76-augustus-gene-0.1	Chromosome Un0137
LOC100554253	maker-scaffold-W292525-augustus-gene-0.2	Chromosome Un0208
PPP6R1	maker-scaffold-W234602-augustus-gene-0.1	Chromosome Un0163
LOC103279913	augustus_masked-scaffold-W353738-processed-gene-0.0	Chromosome Un0087
RBM14	augustus_masked-scaffold-W309787-processed-gene-0.1	Chromosome Un1075
CDK5R1	augustus_masked-scaffold-Wagouti48-processed-gene-0.0	Chromosome Un1272
LOC103279368	maker-scaffold-W290496-augustus-gene-0.1	Chromosome Un0001
DDIT4	augustus_masked-scaffold-W413962-processed-gene-0.0	Chromosome 3

Supplementary Table S9. Inferred copy number for W-linked genes based on female read mapping. Genes without a Z chromosome position did not have a matched Z gametolog.

Gene	Z chromosome position	W chromosome annotation	Copy number
GARS	2,476,541	augustus_masked-scaffold-W225864-processed-gene-0.0	0.86
RBM33	6,652,235	maker-scaffold-W289984-augustus-gene-0.0	0.91
LARP4B	9,429,493	maker-scaffold-Wagouti91-augustus-gene-0.1	0.85
CUL2	13,072,015	maker-scaffold-W187323-augustus-gene-0.0	1.15
EPC1	14,838,677	maker-scaffold-W236610-augustus-gene-0.1	0.95
ZEB1	15,120,182	augustus_masked-scaffold-W328303-processed-gene-0.0	0.98
WAC	16,279,701	maker-scaffold-W328908-augustus-gene-0.1	0.99
TMBIM7P	18,211,983	augustus_masked-scaffold-W279443-processed-gene-0.0	1.18
NMT2	20,364,506	maker-scaffold-Wagouti65-augustus-gene-0.1	1.06
BMI1	24,004,966	augustus_masked-scaffold-W214186-processed-gene-0.0	0.82
PTF1A	24,384,785	maker-scaffold-W214303-augustus-gene-0.1	0.92
UMAD1	27,718,821	maker-scaffold-W335230-augustus-gene-0.1	1.08
SP4	33,937,051	augustus_masked-scaffold-W213628-processed-gene-0.0	1.01
SP4	33,953,392	maker-scaffold-W284381-augustus-gene-0.1	0.93
HOXA1	36,544,550	augustus_masked-scaffold-W339681-processed-gene-0.0	0.71
HOXA2	36,550,838	augustus_masked-scaffold-W339681-processed-gene-0.1	0.75
HOXA6	36,597,885	augustus_masked-scaffold-W246906-processed-gene-0.0	1.07
HOXA9	36,617,150	augustus_masked-scaffold-W332733-processed-gene-0.1	1.07
DAZL	38,445,412	maker-scaffold-W275390-augustus-gene-0.1	0.96
NA	40,973,743	augustus_masked-scaffold-W209805-processed-gene-0.1	1.63
MLV	40,974,268	augustus_masked-scaffold-W286914-processed-gene-0.0	9.38
STT3B	45,446,651	maker-scaffold-Wagouti32-augustus-gene-0.0	0.89
SNRK	46,642,535	augustus_masked-scaffold-W211637-processed-gene-0.0	1.06
LOC106551782	48,075,357	maker-scaffold-W291146-augustus-gene-0.1	1.61
TMEM108	48,228,093	maker-scaffold-W296732-augustus-gene-0.1	1.67
CCR5	48,423,830	augustus_masked-scaffold-W405142-processed-gene-0.0	1.14
CTNNB1	49,525,535	maker-scaffold-W369195-augustus-gene-0.1	1.76
PSMB3	74,103,894	maker-scaffold-W342219-augustus-gene-0.1	1.01
PIP4K2B	74,113,048	augustus_masked-scaffold-W245583-processed-gene-0.0	1.06
CYP2C5	76,177,000	maker-scaffold-W199021-augustus-gene-0.0	0.90
ANKRD17	76,427,468	maker-scaffold-W377123-augustus-gene-0.1	2.56
GPATCH8	77,979,781	augustus_masked-scaffold-W245241-processed-gene-0.0	1.10
FAM171A2	78,127,648	augustus_masked-scaffold-W290328-processed-gene-0.1	1.20
PSMD11	79,118,366	augustus_masked-scaffold-W327146-processed-gene-0.0	0.99
TLK2	79,712,309	maker-scaffold-W350165-augustus-gene-0.1	1.01
UBTF	80,659,852	maker-scaffold-Wagouti18-augustus-gene-0.2	0.97
FBXL20	81,511,122	maker-scaffold-W326727-augustus-gene-0.1	0.98
CDK12	81,609,785	maker-scaffold-Wagouti35-augustus-gene-0.1	0.80
LCA5	82,689,887	maker-scaffold-W396540-augustus-gene-0.1	7.70
MSL1	82,981,183	augustus_masked-scaffold-Wagouti38-processed-gene-0.0	0.98
RARA	83,517,645	maker-scaffold-W315484-augustus-gene-0.0	1.20
STAT5B	86,441,671	augustus_masked-scaffold-Wagouti31-processed-gene-0.0	1.04
KPNB1	87,892,282	maker-scaffold-W235034-augustus-gene-0.1	0.90
SP2	88,396,988	maker-scaffold-Wagouti85-augustus-gene-0.1	1.11
NFE2L1	88,670,286	augustus_masked-scaffold-W234611-processed-gene-0.0	1.08
CBX1	88,702,830	maker-scaffold-Wagouti10-augustus-gene-0.0	1.68
HOXB3	89,380,338	maker-scaffold-Wagouti86-augustus-gene-0.1	1.06
HOXB6	89,440,816	augustus_masked-scaffold-W329503-processed-gene-0.0	1.34
HOXB8	89,459,248	augustus_masked-scaffold-W310848-processed-gene-0.0	1.04
UBE2Z	89,730,244	maker-scaffold-Wagouti54-augustus-gene-0.1	0.96
ZNF652	90,035,554	maker-scaffold-W279164-augustus-gene-0.1	1.18
SPOP	90,532,934	maker-scaffold-Wagouti63-augustus-gene-0.1	0.92
KAT7	90,759,908	maker-scaffold-Wagouti34-augustus-gene-0.1	0.97
HIST1H1A	91,479,689	augustus_masked-scaffold-W292525-processed-gene-0.1	1.51
COL1A1	91,497,833	maker-scaffold-W361806-augustus-gene-0.1	2.05
HDAC5	92,334,440	augustus_masked-scaffold-W278334-processed-gene-0.0	0.97

SCAF1	92,837,626	maker-scaffold-W308895-augustus-gene-0.1	1.10
RRAS	92,879,514	maker-scaffold-W287227-augustus-gene-0.0	1.09
PRR12	92,897,746	maker-scaffold-W297391-augustus-gene-0.1	1.17
AP2A1	93,239,319	maker-scaffold-W244580-augustus-gene-0.1	1.00
SNRNP70	93,458,083	augustus_masked-scaffold-Wagouti74-processed-gene-0.0	1.06
PPFIA3	93,527,902	augustus_masked-scaffold-Wagouti36-processed-gene-0.0	0.87
TRIM28	95,035,682	maker-scaffold-W327090-augustus-gene-0.1	1.06
ZSCAN16	98,343,344	maker-scaffold-W355712-augustus-gene-0.1	1.87
ZNF219	98,510,960	augustus_masked-scaffold-W225694-processed-gene-0.0	1.21
ZNF268	98,535,594	augustus_masked-scaffold-W243873-processed-gene-0.0	1.08
MFAP4	99,720,703	maker-scaffold-Wagouti68-augustus-gene-0.1	0.85
ERV3-1	99,846,619	augustus_masked-scaffold-W225890-processed-gene-0.0	6.11
PRSS27	100,149,893	maker-scaffold-W225852-augustus-gene-0.1	0.94
PAQR4	100,400,887	maker-scaffold-W207139-augustus-gene-0.1	0.95
APOBR	100,797,148	augustus_masked-scaffold-W276738-processed-gene-0.0	0.93
ATP2A1	100,982,867	augustus_masked-scaffold-Wagouti33-processed-gene-0.0	1.08
SH2B1	101,055,049	augustus_masked-scaffold-W229965-processed-gene-0.0	1.00
TBC1D17	101,342,258	maker-scaffold-W233879-augustus-gene-0.1	1.04
GYS1	101,438,445	augustus_masked-scaffold-W215996-processed-gene-0.0	1.10
FTL	101,478,377	maker-scaffold-W328357-augustus-gene-0.2	2.81
DNAAF3	102,286,382	augustus_masked-scaffold-W313163-processed-gene-0.0	0.88
PTPRH	102,356,675	maker-scaffold-W239731-augustus-gene-0.2	1.17
PPP6R1	102,450,425	augustus_masked-scaffold-W290532-processed-gene-0.0	0.96
HSPBP1	102,507,380	maker-scaffold-Wagouti53-augustus-gene-0.1	1.04
TMEM150B	102,645,603	maker-scaffold-W347648-augustus-gene-0.0	0.80
EPS8L1	102,988,692	maker-scaffold-W292571-augustus-gene-0.0	1.19
KCNJ14	103,238,334	maker-scaffold-W314154-augustus-gene-0.1	1.14
CYTH2	103,245,655	augustus_masked-scaffold-W229997-processed-gene-0.0	1.07
TLR13	103,277,956	maker-scaffold-W283781-augustus-gene-0.1	1.04
LMTK3	103,359,411	augustus_masked-scaffold-Wagouti9-processed-gene-0.0	1.17
LMTK3	103,359,411	maker-scaffold-Wagouti9-augustus-gene-0.2	0.75
CACNG7	103,482,560	augustus_masked-scaffold-Wagouti22-processed-gene-0.1	1.31
CACNG8	103,538,585	augustus_masked-scaffold-W278382-processed-gene-0.0	0.88
CCDC114	103,736,700	maker-scaffold-W211154-augustus-gene-0.1	1.05
PRPF31	104,144,386	maker-scaffold-W309302-augustus-gene-0.1	1.02
TFPT	104,158,398	maker-scaffold-Wagouti12-augustus-gene-0.1	1.00
OSCAR	104,174,068	maker-scaffold-W285225-augustus-gene-0.1	1.11
SHISA7	104,290,908	maker-scaffold-W207421-augustus-gene-0.1	1.07
UBE2S	104,335,372	maker-scaffold-Wagouti90-augustus-gene-0.1	3.89
RCVRN	104,360,810	augustus_masked-scaffold-W225900-processed-gene-0.0	1.12
RASIP1	104,548,230	maker-scaffold-W210503-augustus-gene-0.0	0.92
IZUMO1	104,686,082	maker-scaffold-W345695-augustus-gene-0.0	1.10
SULT2B1	104,763,789	maker-scaffold-W278386-augustus-gene-0.0	0.94
ISOC2	104,936,777	maker-scaffold-W327885-augustus-gene-0.0	0.95
MYH11	104,952,866	augustus_masked-scaffold-W286112-processed-gene-0.1	1.01
EPN1	105,074,770	augustus_masked-scaffold-W246072-processed-gene-0.0	0.88
U2AF2	105,112,978	augustus_masked-scaffold-W311918-processed-gene-0.0	2.14
ZNF726	105,206,563	augustus_masked-scaffold-W236876-processed-gene-0.1	1.26
SACS	105,235,849	augustus_masked-scaffold-W353738-processed-gene-0.1	0.99
H4C1	105,318,166	augustus_masked-scaffold-W292525-processed-gene-0.0	0.98
TBRG1	105,389,554	augustus_masked-scaffold-W315309-processed-gene-0.0	1.16
CORO1A	105,450,385	maker-scaffold-W331055-augustus-gene-0.1	0.92
GDPD3	105,512,448	augustus_masked-scaffold-Wagouti22-processed-gene-0.0	0.98
YPEL3	105,531,125	maker-scaffold-W211880-augustus-gene-0.0	0.97
ALDOA	105,582,679	augustus_masked-scaffold-W210200-processed-gene-0.0	1.31
MVP	105,631,040	maker-scaffold-Wagouti3-augustus-gene-0.1	0.93
MAZ	105,680,423	augustus_masked-scaffold-W321164-processed-gene-0.0	0.94
PRRT2	105,690,065	augustus_masked-scaffold-W230767-processed-gene-0.0	0.82
CDIPT	105,710,861	augustus_masked-scaffold-W311991-processed-gene-0.0	1.01

KIF22	105,835,326	maker-scaffold-W327953-augustus-gene-0.1	0.91
PAGR1	105,856,879	augustus_masked-scaffold-W349507-processed-gene-0.0	1.07
ASPHD1	105,865,513	maker-scaffold-W349507-augustus-gene-0.3	1.09
SEZ6L2	105,873,042	maker-scaffold-W244755-augustus-gene-0.1	0.99
INO80E	105,967,889	maker-scaffold-W274364-augustus-gene-0.1	1.20
GJB2	106,198,960	augustus_masked-scaffold-W283743-processed-gene-0.0	1.13
THOC6	106,257,774	maker-scaffold-W277668-augustus-gene-0.0	1.05
HCFC1R1	106,267,800	maker-scaffold-W238660-augustus-gene-0.2	1.45
RAB35	106,273,569	maker-scaffold-W238660-augustus-gene-0.1	0.82
TBC1D10B	106,457,061	augustus_masked-scaffold-W285251-processed-gene-0.0	1.15
CA4	106,793,422	augustus_masked-scaffold-W292519-processed-gene-0.0	2.41
LOC106538011	–	augustus_masked-scaffold-W198575-processed-gene-0.0	1.23
KANSL1	–	augustus_masked-scaffold-W210266-processed-gene-0.0	1.22
PRR12	–	augustus_masked-scaffold-W211159-processed-gene-0.0	1.05
LOC106547512	–	augustus_masked-scaffold-W234755-processed-gene-0.1	2.00
LOC106548453	–	augustus_masked-scaffold-W244348-processed-gene-0.0	1.04
ERV3-1	–	augustus_masked-scaffold-W245263-processed-gene-0.0	6.26
HA1F	–	augustus_masked-scaffold-W278059-processed-gene-0.0	5.93
SOX8	–	augustus_masked-scaffold-W278460-processed-gene-0.0	1.51
LOC106548453	–	augustus_masked-scaffold-W279081-processed-gene-0.0	1.60
ZNF319	–	augustus_masked-scaffold-W283894-processed-gene-0.0	1.33
GYS1	–	augustus_masked-scaffold-W284440-processed-gene-0.0	1.09
NA	–	augustus_masked-scaffold-W284442-processed-gene-0.0	4.78
NA	–	augustus_masked-scaffold-W285958-processed-gene-0.0	3.49
RBM14	–	augustus_masked-scaffold-W290042-processed-gene-0.0	4.19
RBM14	–	augustus_masked-scaffold-W290085-processed-gene-0.0	4.82
FAM171A2	–	augustus_masked-scaffold-W290328-processed-gene-0.0	1.04
LOC106547512	–	augustus_masked-scaffold-W291562-processed-gene-0.0	1.34
LOC106548453	–	augustus_masked-scaffold-W291653-processed-gene-0.0	0.98
H2-Q9	–	augustus_masked-scaffold-W298664-processed-gene-0.0	15.59
ERV3-1	–	augustus_masked-scaffold-W309347-processed-gene-0.0	4.05
LOC106538011	–	augustus_masked-scaffold-W309787-processed-gene-0.0	1.28
CACNA2D3	–	augustus_masked-scaffold-W309787-processed-gene-0.1	4.94
LOC106548453	–	augustus_masked-scaffold-W309821-processed-gene-0.0	1.37
PRR12	–	augustus_masked-scaffold-W312358-processed-gene-0.1	0.92
LOC106538011	–	augustus_masked-scaffold-W313442-processed-gene-0.0	1.25
ERV3-2	–	augustus_masked-scaffold-W315963-processed-gene-0.0	6.19
ERV3-1	–	augustus_masked-scaffold-W327813-processed-gene-0.0	10.25
SNRK	–	augustus_masked-scaffold-W339755-processed-gene-0.0	0.98
SOX8	–	augustus_masked-scaffold-W339823-processed-gene-0.0	1.66
ERV3-1	–	augustus_masked-scaffold-W340974-processed-gene-0.0	3.20
LOC106548478	–	augustus_masked-scaffold-W349682-processed-gene-0.0	1.42
SACS	–	augustus_masked-scaffold-W353738-processed-gene-0.0	1.10
LOC106538011	–	augustus_masked-scaffold-W366597-processed-gene-0.0	1.14
MDFI	–	augustus_masked-scaffold-W391774-processed-gene-0.0	1.12
DDIT4L	–	augustus_masked-scaffold-W413962-processed-gene-0.0	2.01
LOC106548453	–	augustus_masked-scaffold-W462876-processed-gene-0.0	1.16
LOC106547512	–	augustus_masked-scaffold-Wagouti23-processed-gene-0.0	1.36
ERV3-1	–	augustus_masked-scaffold-Wagouti42-processed-gene-0.0	8.71
CDK5R1	–	augustus_masked-scaffold-Wagouti48-processed-gene-0.0	0.98
CDK12	–	augustus_masked-scaffold-Wagouti67-processed-gene-0.0	0.81
CDK12	–	augustus_masked-scaffold-Wagouti67-processed-gene-0.1	0.93
CDK12	–	augustus_masked-scaffold-Wagouti67-processed-gene-0.2	1.18
ERV3-1	–	augustus_masked-scaffold-Wagouti87-processed-gene-0.0	3.30
LOC106551782	–	maker-scaffold-W214192-augustus-gene-0.1	1.46
LOC106551782	–	maker-scaffold-W225851-augustus-gene-0.1	1.64
PPP6R1	–	maker-scaffold-W234602-augustus-gene-0.1	1.07
EPC1	–	maker-scaffold-W283905-augustus-gene-0.1	0.79
NA	–	maker-scaffold-W290496-augustus-gene-0.1	2.48

NA	–	maker-scaffold-W291004-augustus-gene-0.1	6.24
TRIM28	–	maker-scaffold-W296260-augustus-gene-0.1	0.98
LOC106551782	–	maker-scaffold-W310586-augustus-gene-0.1	1.36
RBM33	–	maker-scaffold-W313771-augustus-gene-0.1	0.84
ERV3-1	–	maker-scaffold-W315223-augustus-gene-0.1	3.27
SEZ6L2	–	maker-scaffold-W315725-augustus-gene-0.1	1.03
LOC106549959	–	maker-scaffold-W327100-augustus-gene-0.1	1.10
NA	–	maker-scaffold-W328357-augustus-gene-0.1	5.24
LOC106551782	–	maker-scaffold-W331333-augustus-gene-0.1	1.27
C5	–	maker-scaffold-W361845-augustus-gene-0.1	0.69
LOC106538011	–	maker-scaffold-W376851-augustus-gene-0.1	1.88
AADACL4	–	maker-scaffold-W382211-augustus-gene-0.1	3.05
SLC22A7	–	maker-scaffold-W404072-augustus-gene-0.1	1.09
ERV3-1	–	maker-scaffold-W48-augustus-gene-0.1	1.94
UBTF	–	maker-scaffold-Wagouti18-augustus-gene-0.3	0.91
PRR12	–	maker-scaffold-Wagouti58-augustus-gene-0.1	1.26
RT1-B	–	maker-scaffold-Wagouti70-augustus-gene-0.0	22.52
TMEM143	–	maker-scaffold-Wagouti76-augustus-gene-0.1	0.99
VWA5A	–	maker-scaffold-Wagouti77-augustus-gene-0.1	3.66
NA	–	maker-scaffold-Wagouti84-augustus-gene-0.2	1.28
MLV	–	augustus_masked-scaffold-W236876-processed-gene-0.0	73.51
NA	–	augustus_masked-scaffold-W278718-processed-gene-0.0	1.35
NA	–	augustus_masked-scaffold-W278741-processed-gene-0.0	1.19
NA	–	augustus_masked-scaffold-W278965-processed-gene-0.0	0.87
NA	–	augustus_masked-scaffold-W280125-processed-gene-0.1	2.18
NA	–	augustus_masked-scaffold-W283802-processed-gene-0.0	1.09
NA	–	augustus_masked-scaffold-W284345-processed-gene-0.0	1.54
NA	–	augustus_masked-scaffold-W290901-processed-gene-0.0	1.65
NA	–	augustus_masked-scaffold-W309211-processed-gene-0.0	1.21
NA	–	augustus_masked-scaffold-W315149-processed-gene-0.0	1.22
NA	–	augustus_masked-scaffold-W332734-processed-gene-0.0	0.57
NA	–	augustus_masked-scaffold-W339702-processed-gene-0.0	2.14
NA	–	augustus_masked-scaffold-W343715-processed-gene-0.0	1.60
MLV	–	augustus_masked-scaffold-W368604-processed-gene-0.0	11.91
NA	–	augustus_masked-scaffold-W368988-processed-gene-0.0	1.31
NA	–	augustus_masked-scaffold-W381771-processed-gene-0.0	2.29
NA	–	augustus_masked-scaffold-Wagouti24-processed-gene-0.0	1.43
NA	–	augustus_masked-scaffold-Wagouti36-processed-gene-0.1	1.92
NA	–	augustus_masked-scaffold-Wagouti50-processed-gene-0.0	1.15
NA	–	maker-scaffold-W210647-augustus-gene-0.1	0.88
NA	–	maker-scaffold-W225917-augustus-gene-0.0	4.97
NA	–	maker-scaffold-W292525-augustus-gene-0.2	1.91
NA	–	maker-scaffold-W331308-augustus-gene-0.1	6.18
NA	–	maker-scaffold-W359059-augustus-gene-0.0	21.26
NA	–	maker-scaffold-Wagouti32-augustus-gene-0.1	0.91
